# Construction of Host Plant Insect‐Resistance Mutant Library by High‐Throughput CRISPR/Cas9 System and Identification of A Broad‐Spectrum Insect Resistance Gene

**DOI:** 10.1002/advs.202306157

**Published:** 2023-11-30

**Authors:** Lin Sun, Muna Alariqi, Yaxin Wang, Qiongqiong Wang, Zhongping Xu, Muhammad Naeem Zafar, Guangqin Yang, Ruoyu Jia, Amjad Hussain, Yilin Chen, Xiao Ding, Jiawei Zhou, Guanying Wang, Fuqiu Wang, Jianying Li, Jiawei Zou, Xiangqian Zhu, Lu Yu, Yiwen Sun, Sijia Liang, Fengjiao Hui, Luo Chen, Weifeng Guo, Yanqin Wang, Huaguo Zhu, Keith Lindsey, Xinhui Nie, Xianlong Zhang, Shuangxia Jin

**Affiliations:** ^1^ Hubei Hongshan Laboratory National Key Laboratory of Crop Genetic Improvement Huazhong Agricultural University Wuhan Hubei 430070 P. R. China; ^2^ Institute of Industrial Crops Shandong Academy of Agricultural Sciences Jinan Shandong 250100 China; ^3^ Department of Agronomy and Pastures, Faculty of Agriculture Sana’a University Sana’a Yemen; ^4^ Xinjiang Production and Construction Corps Key Laboratory of Protection and Utilization of Biological Resources in Tarim Basin Tarim University Alaer Xinjiang 843300 China; ^5^ College of Biology and Agricultural Resources Huanggang Normal University Huanggang Hubei 438000 China; ^6^ Department of Biosciences Durham University Durham DH1 3LE UK; ^7^ Key Laboratory of Oasis Ecology Agricultural of Xinjiang Bingtuan Agricultural College Shihezi University Shihezi Xinjiang China

**Keywords:** cotton, CRISPR/Cas9, GhEPS15, GhMLP423, high‐throughput genome editing, plant‐insect interaction

## Abstract

Insects pose significant challenges in cotton‐producing regions. Here, they describe a high‐throughput CRISPR/Cas9‐mediated large‐scale mutagenesis library targeting endogenous insect‐resistance‐related genes in cotton. This library targeted 502 previously identified genes using 968 sgRNAs, generated ≈2000 T0 plants and achieved 97.29% genome editing with efficient heredity, reaching upto 84.78%. Several potential resistance‐related mutants (10% of 200 lines) their identified that may contribute to cotton‐insect molecular interaction. Among these, they selected 139 and 144 lines showing decreased resistance to pest infestation and targeting **m**ajor **l**atex‐like **p**rotein 423 (*GhMLP423*) for in‐depth study. Overexpression of *GhMLP423* enhanced insect resistance by activating the plant systemic acquired resistance (SAR) of salicylic acid (SA) and *pathogenesis‐related* (*PR*) genes. This activation is induced by an elevation of cytosolic calcium [Ca^2+^]_cyt_ flux eliciting reactive oxygen species (ROS), which their demoted in *GhMLP423* knockout (CR) plants. Protein‐protein interaction assays revealed that GhMLP423 interacted with a human epidermal growth factor receptor substrate15 (EPS15) protein at the cell membrane. Together, they regulated the systemically propagating waves of Ca^2+^ and ROS, which in turn induced SAR. Collectively, this large‐scale mutagenesis library provides an efficient strategy for functional genomics research of polyploid plant species and serves as a solid platform for genetic engineering of insect resistance.

## Graphical abstract

A high‐throughput screening strategy for cotton endogenous insect‐resistance‐related genes is established by a CRISPR/Cas9‐mediated large‐scale mutant library. It identifies promising genes related to plant innate insect‐resistance, including **m**ajor **l**atex‐like **p**rotein 423 (*GhMLP423*). *GhMLP423* enhanced plant immunity to chewing and sap‐sacking insects via the induction of *GhEPS15* that initiates the systemic acquired resistance elicited by the systemically propagating waves of Ca^2+^‐mediated ROS signaling.

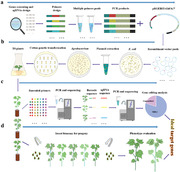

## 1 Introduction

Functional genomics research relies on the screening of a large number of mutants; however, the low rate of natural mutations cannot meet the advancement of research needs. Several traditional experimental methods are used for plant mutation breeding, like physical radiation and chemical mutagenesis techniques.^[^ ^1^ ^]^ In addition, *Agrobacterium*‐mediated T‐DNA integration strategy has revolutionized plant functional genomics,^[^ ^2^ ^]^ by providing a rich resource of mutants to identify gene function and link genotype to phenotypes in many plant species.^[^ ^3^ ^]^ However, T‐DNA insertion is randomly inserted in the genome and often integrates in intergenic or non‐coding regions which may generate undetectable phenotypes.^[^ ^4^ ^]^ This phenomenon can be largely found in polyploid plant species due to gene redundancy.^[^ ^5^ ^]^ Therefore, it is an arduous task to create a large‐ or genome‐scale loss‐of‐function mutation library for polyploid plant species with complex genomes.

In recent years, Clustered Regularly Interspaced Short Palindromic Repeats (CRISPR) has become a powerful and universal tool for functional genomics. So far, the CRISPR/Cas9 system has been successfully applied to major crops.^[^ ^6^ ^]^ In cotton, our lab has established several efficient and precise CRISPR/Cas variants with high editing efficiency and low off‐target effects.^[^ ^7^ ^]^ Due to its ability for high efficiency, the CRISPR/Cas9 system has been recruited to generate large‐scale mutagenesis libraries and has become a common technique in human and animal cell culture.^[^ ^8^ ^]^ However, there are only a few reports regarding high‐throughput CRISPR/Cas9 screening in plants due to the labor‐ and time‐intensive genetic transformation process.^[^ ^9^ ^]^ Fortunately, our established CRISPR/Cas9 genome editing system in cotton makes it possible to build a large‐scale mutant library for cotton genomics research.

Cotton is a major economic crop, and cotton fiber is a globally leading textile material.^[^ ^10^ ^]^ With the wide adoption of transgenic cotton, *Cry* genes encoding *Bacillus thuringiensis* (*BT*) toxins have successfully controlled two major lepidopteran pests, *Helicoverpa armigera* and *Pectinophora* gossy*piella*.^[^ ^11^ ^]^ However, the current commercial transgenic *BT* cotton (Cry IAc/Ab) has no insecticidal effects on most sap‐sucking insects, such as whiteflies, aphids, and lygus.^[^ ^12^ ^]^ In recent years, sap‐sucking insects have emerged as the most destructive pests in cotton fields in China. For instance, according to a report from the Plant Protection Station in 2019, aphid infestation affected 442000 hectares of cotton fields, accounting for around 20% of the total cotton cultivation area in Xinjiang region. Since 2015, we have used multiple 'omics strategies (transcriptomics, proteomics, and metabolomics) to investigate cotton host defense system against sap‐sucking insects and to identify several hundred of genes related to cotton host resistance to insect infestation.^[^ ^13^ ^]^ The identification of endogenous genes for insect‐resistance breeding is a promising strategy in cotton, which could be complementary to the use of *BT* cotton. Therefore, an efficient and reliable strategy for the high‐throughput functional analysis of genes is desirable for cotton functional genomics.

A successful plant defense system depends on the ability to quickly transmit sensing signals from the initial site of occurrence to the entire plant in response to external stimuli events, such as wounding or pathogen infection.^[^ ^14^ ^]^ Several studies highlighted the role of some rapid plant systemic signals, like calcium (Ca^2+^), reactive oxygen species (ROS), hydraulic waves and electric waves, in activating plant defense to various biotic and abiotic stressors.^[^ ^15^ ^]^ Ca^2+^ and ROS waves are originally proposed to interact together with electrical signals to constitute a major part of plant rapid signaling networks and cell‐to‐cell communication.^[^ ^16^ ^]^ As a second messenger that is elevated in response to wounding, cytosolic Ca^2+^ ([Ca^2+^]_cyt_) is the key player in initiating plant signaling networks that support both local and systemic defense responses.^[^ ^17^ ^]^ The oxidative burst is one of the most immediate stimuli‐induced defense responses that is triggered by the elevation of [Ca^2+^]_cyt_ at the sensing tissues and together propagate to distal tissues to induce downstream systematic responses, including changes in protein levels, biochemical activities, metabolite production, phytohormone induction, and even reprogram gene expression to produce antimicrobial or anti‐insect compounds, such as pathogenesis‐related (PR) proteins.^[^ ^18^ ^]^ Even though it has been shown that several signals take part in the induction of plant systemic responses, many questions about their mode of operation, propagating paths, and integration still need to be resolved.

Major latex‐like proteins (MLPs) were primarily recognized as laticifer‐specific binding proteins in the latex of *Papaver somniferum*.^[^ ^19^ ^]^ MLPs are one of the protein subclasses of the Bet v 1 family, along with PR10 proteins, cytokinin‐specific binding proteins and norcoclaurine synthases.^[^ ^20^ ^]^ They have been identified in many plant species, such as *Arabidopsis*,^[^ ^21^ ^]^ cotton^[^ ^22^ ^]^ and tobacco.^[^ ^23^ ^]^ The expression of *MLPs* genes is associated with plant defense responses to drought stress,^[^ ^24^ ^]^ pathogens and wounding.^[^ ^25^ ^]^ The upregulation of the *MLP*‐Patty Green (*MLP‐PG1*) gene induced the expression of *PR‐2* and *PR‐5* genes, which resulted in enhanced resistance against pathogens,^[^ ^26^ ^]^ however, the mechanism underlying the induction of *PR* genes is still elusive.

In this study, we describe a method for screening endogenous insect‐resistant genes by establishing a CRISPR/Cas9‐mediated high‐throughput mutant library in cotton. A plasmid vector library containing 969 single guide RNAs (sgRNAs) was used to generate a mutant population targeting 502 cotton‐endogenous genes mainly involved in cotton host resistance against insect infestation. More than 2000 individual T0 mutants have been obtained, and 200 T1 lines were randomly selected and subjected to insect bioassays in greenhouse and field conditions. From this library, *GhMLP423* was identified as a broad‐spectrum insect‐resistant gene that enhanced plant defense via initiating systemic acquired resistance (SAR) of salicylic acid (SA) and *PR* genes elicited by the systemically propagating waves of Ca^2+^‐mediated ROS signaling.

## 2 Results

### 2.1 Construction and Evaluation of a Pooled sgRNA Library

**Figure 1:**
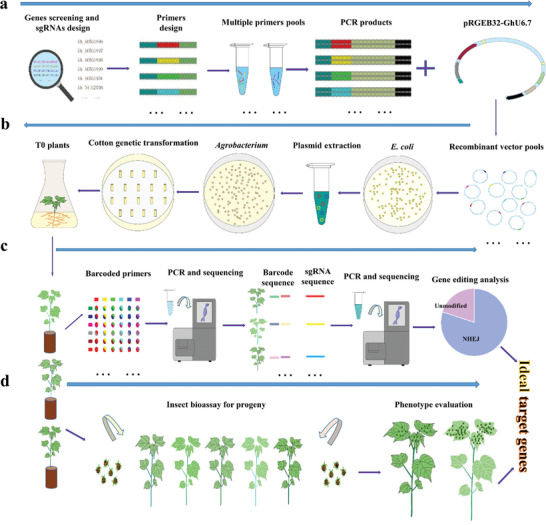
Schematic diagram of high‐throughput mutant library construction for CRISPR/Cas9 system in cotton. a) The construction of pooled sgRNAs library, including gene and sgRNA screening, primer designing, primers pooling, and PCR amplification. b) Genetic transformation of cotton was carried out using pooled *Agrobacterium* strains. A number of small‐size plasmid libraries were propagated in *E. coli* and plasmids were extracted and mixed in equal quantities to transform to *Agrobacterium*. A large number of *Agrobacterium* colonies were pooled for cotton transformation. c) Barcode technology and high‐throughput sequencing were used to identify the editing sites and editing profiles in different samples. Regenerated T0 plants usually have multiple editing sites (distinguished by colors and outlines). d) Phenotype detection and insect bioassays were combined with DNA sequencing to identify candidate genes for future research.

In order to identify the potential insect‐resistant‐related genes, we analyzed a large number of differentially expressed genes (DEGs) in insect‐resistant and ‐susceptible genotypes from our previous transcriptome studies.^[^ ^27^ ^]^ Out of which, 502 genes were enriched in stress‐related responses and signaling pathways (Figure S1, Table S1, Supporting Information). To functionally identify this number of genes using the conventional CRISPR/Cas9 method, a huge number of vectors need to be constructed, which is laborious and time‐consuming. Therefore, we developed a high‐throughput genome editing method to construct multiple vectors targeting 502 genes at once (**Figure** **1**). Initially, a preliminary experiment was conducted to verify the viability of the proposed pooled vector library. A small library was constructed containing 40 sgRNAs targeting 20 genes; each gene was targeted by two sgRNAs. Oligo sgRNAs were ligated to homologous primer sequences to the linear ends of the CRISPR/Cas9 plasmid *pRGEB32‐GhU6.7*, mixed in equal amounts, assembled into the *pRGEB32‐GhU6.7* after PCR amplification, and subsequently, 100 randomly selected *E. coli* clones were harvested and identified via high‐throughput sequencing. Results revealed that 34 sgRNAs were identified that completely covered all 20 target genes (Table S2, Supporting Information). The preliminary experimental results ensured efficient coverage of the vector library with no obvious bias, indicating that this method can be used to construct a large‐scale vector library. Through genome‐wide comparative analysis, a total of 969 highly distinct sgRNAs were designed to target the 502 genes; every gene was targeted by at least one sgRNA (Table S3, Supporting Information). The final pooled vector library was constructed by the aforementioned method using 969 pair primers that were divided into 24 subprimer‐pools. Each pool was used independently for PCR amplification, vector construction, and plasmid extraction. Ultimately, the 24 sub‐libraries were mixed in equal amounts to obtain a pooled plasmid vector library ready for the *Agrobacterium‐*mediated transformation. More than 10000 *Agrobacterium* clones were collected for cotton genetic transformation (Figure S2, Supporting Information).

### 2.2 Identification and Characterization of Mutants in the CRISPR/Cas9 Library

The first step to verifying the efficiency of the pooled vector library is to identify the target sites of sgRNAs located in the genome of the generated plants. More than 2000 independent T0 transgenic plants were regenerated in this mutant library (**Table** **1**). To test the efficiency of *Agrobacterium*‐mediated transformation, a PCR‐based barcoding library was constructed to identify the sgRNAs in 1380 T0 plants. A total of 44 primers (Table S4, Supporting Information) containing different barcodes at the 5′ ends were designed to encode the target sequences in 384 plant samples at once, according to a previous report.^[^ ^28^ ^]^ A total of 555 different sgRNA sequences targeting 412 genes were identified, representing 82.07% of the total target genes. Analysis of 1380 T0 plants showed that 1079 plants (78.19%) harbored one or more sgRNA sequences, among which 1015 plants contained one sgRNA, 58 plants contained two different sgRNAs, 4 plants contained three sgRNAs, and only 2 plants contained multiple sgRNAs (more than 3 sgRNAs). These results suggest that 94.07% of T0 plants harbored only one sgRNA sequence (**Table** **2**), which is consistent with the conclusion that the T‐DNA insertions in plant genome mainly consist of a low copy number (1−2 copies).

**Table 1:** Summary of the mutant library size and sgRNAs coverage rate.

Mutant library size	The number of tested samples	sgRNAs numbers in T0 plants	Number of T0 plants with sgRNAs	Ratio of T0 plants with sgRNAs
2000+ T0 plants	1380	Negative	301	21.81%
		1 sgRNA	1015	73.55%
		2 sgRNAs	58	4.20%
		3 sgRNAs	4	0.29%
		3+ sgRNAs	2	0.14%

**Table 2:** Editing frequency of 12 T1 lines compared to T0 parents.

Mutant line	Editing frequency of T0 plant	Editing frequency of T1 plants		
Line 175	0.67%	99.48%	93.46%	94.11%
Line 378	4.21%	57.94%	5.20%	31,44%
Line 139	11.17%	61.31%	79.03%	46.07%
Line 537	48.94%	76.82%	98.03%	91.62%
Line 215	49.42%	71.91%	93.59%	95.62%
Line 187	49.43%	98.93%	99.52%	96.50%
Line 70	50.84%	98.49%	93.28%	87.81%
Line 127	57.96%	95.85%	99.50%	99.68%
Line 422	58.21%	97.17%	95.16%	80.58%
Line 77	58.91%	99.32%	58.62%	99.02%
Line 56	60.01%	100%	100%	‐
Line 36	62.31%	94.88%	97.91%	95.81%

**Figure 2:**
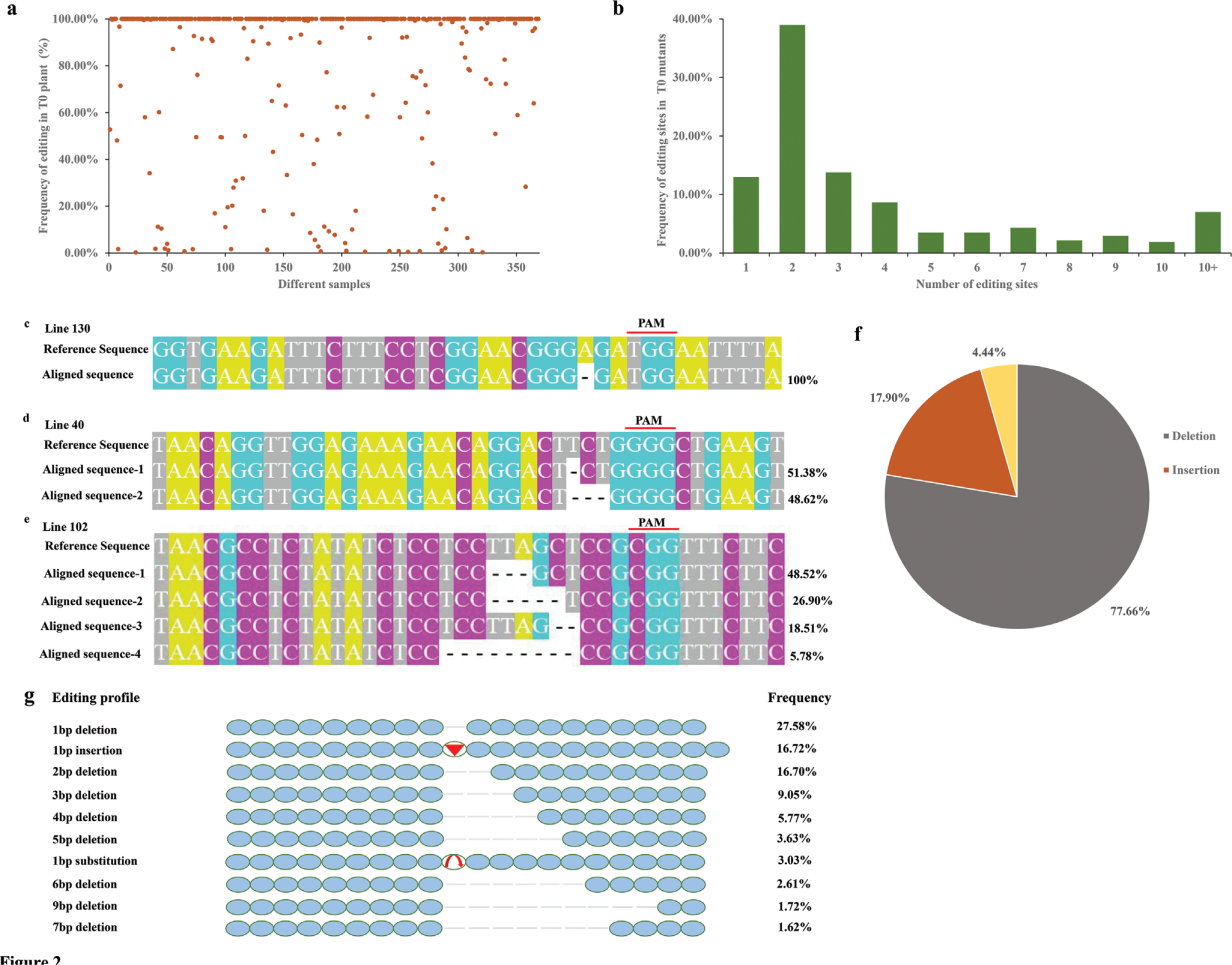
Statistics of editing frequency and the number of editing types produced by CRISPR/Cas9 in cotton. a) Editing frequency statistics of 369 T0 plants. b) Statistics on the number of editing types produced by genome editing in each regenerated plant. c,d,e) Editing genotypes of Line 130, Line 40, and Line 102 compared to the reference sequences GGTGAAGATTTCTTTCCTCGGAACGGGAGATGGAATTTTA, TAACAGGTTGGAGAAAGAACAGGACTTCTGGGGCTGAAGT and TAACGCCTCTATATCTCCTCCTTAGCTCCGCGGTTTCTTC, respectively. f) The overall distribution of insertions, substitutions and deletions. g) The most common ten editing types in the constructed library.

After the determination of the target sites of each plant, the editing profile of 400 independent T0 plants was further analyzed by high‐throughput sequencing. After excluding sequencing errors and mismatches by PCR amplification, a total of 369 effective edited plants were selected for further analyses. The frequency of gene editing >1% (edited reads/total reads from high‐throughput sequencing) was considered authentic editing, whereas samples with a gene‐editing frequency of <1% were regarded as non‐edited samples. Accordingly, results showed that effective genome editing (>1%) reached up to 97.29% (359 out of 369 plants). Of these, 75.61% (279 out of 369) plants showed a highly efficient target mutation rate occurring in more than 80%, and these mutations were mostly homozygous (**Figure** **2**a). To verify the high‐throughput sequencing data, 10 T0 plants were randomly selected for Sanger sequencing, and results of the two sequencing methods were strictly matched, demonstrating that the high‐throughput sequencing is reliable and effective (Figure S3, Supporting Information). Further, the number of editing types was calculated, and results showed that only one editing site was detected in 48 plants (13.00%), while the largest proportion containing two editing sites was detected in 144 plants (39.02%). Plants with multiple mutations (more than two editing sites) accounted for 47.98%, and only 26 plants (7.05%) obtained up to 10 editing sites (Figure 2b). Statistics of the number of editing sites indicate that most of the plants contain multiple editing sites in the same target gene, ensuring complete knockout of these genes. Three typical types of genome editing profiles are illustrated in Figure 2c–e, with examples of single‐site mutation (Line 130), double‐site mutation (Line 40), and multiple‐site mutation (Line 102).

The double‐stranded break (DSB) sites generated by Cas9 endonuclease are mostly repaired by the error‐prone NHEJ pathway, resulting in multiple types of editing profiles, like short fragment insertion, deletion or single base substitution. Deletion of short DNA constitutes the largest proportion of edits (77.66%) in this mutant library, and the majority of these deletions are mainly small fragments (1‐4 bp in length), while larger deletions are rarely verified, in which deletions larger than 30 bp accounted for 0.0049% and only 0.0002% of the deletions exceeded 100 bp (Figure 2f, Figure S4a, Supporting Information). Short insertions were also observed, accounting for 17.90%, which are dominantly 1 bp insertion (Figure 2f, Figure S4b, Supporting Information). Substitutions with 1–4 bp took place in this mutation library as well and accounted for the lowest proportion (4.44%) (Figure 2f, Figure S4c, Supporting Information). The ten most common editing types are listed in Figure 2g.

### 2.3 Complex Inheritance Patterns of Targeted Genome Editing Induced by CRISPR/Cas9

The efficient editing described above demonstrates the feasibility of our CRISPR/Cas9 high‐throughput mutation strategy. In further analyses, we investigated the heritability of editing from T0 to T1 generations. The editing profiles of T1 plants were determined and compared to the corresponding T0 parents. If the editing type of T1 progenies was identical to that of T0, it was considered heritable. Seeds of 100 independent T1 lines were grown in the greenhouse, and three randomly selected plants from each T1 line were used for heredity analysis. The genetic analysis of 275 T1 plants revealed that the vast majority of progeny plants contained the same mutation type as identified in T0 parental plants. The editing heritability ranged from 0% to 100%, with an average of 84.78% (Figure S5a, Supporting Information). Among these 275 plants, 89 (32.36%) presented faithful editing to their T0 parents with a heritability of 100%. In some lines, different types of editing were observed in T1‐positive plants from T0‐generation, among them 23 (8.36%) plants showed novel editing sites. The newly generated editing indicates that Cas9 nucleases can actively edit genes across generations. It was also observed that, among the descendants of 100 tested plants, the editing rate of all T1 plants was significantly improved. It was surprising to find that the editing of line 175 in T0 obtained a low editing efficiency of 0.67%; however, in the three T1 tested plants of line 175, editing efficiency increased up to 99.48%, 93.46% and 94.11% (Table 2). The improved editing rate in the T1 generation indicates that positive T0 plants (containing the T‐DNA insertion/CRISPR/Cas9 unit) with a low editing rate can still be used to generate desired phenotypes in the offspring with elevated editing efficiency.

Although the overall gene editing frequency at target loci was high, the genetic basis of some lines was unclear. For example, line 24 contained three different editing sites, where every plant of the tested offspring faithfully inherited one or more editing sites from its parental line. On the other hand, a new editing site with complicated genetic rules occurred in the offspring plants (Figure S5b, Supporting Information). Taking line 552 as another example, three editing sites were detected in the T0 generation, with an editing rate of 67%. However, the editing type and frequency of the three T1 progenies were unexpected, and the new editing sites produced in plants T1‐2 and T1‐3 were not inherited from T0 at all (Figure S5c, Supporting Information). These special genetic models are different from the known Mendelian segregation, which might be due to the presence of multiple alleles in the cotton genome, incomplete editing, or the persistent activity of the Cas9 endonuclease.

### 2.4 Rapid Screening for Potential Insect Resistant‐Related Genes

**Figure 3:**
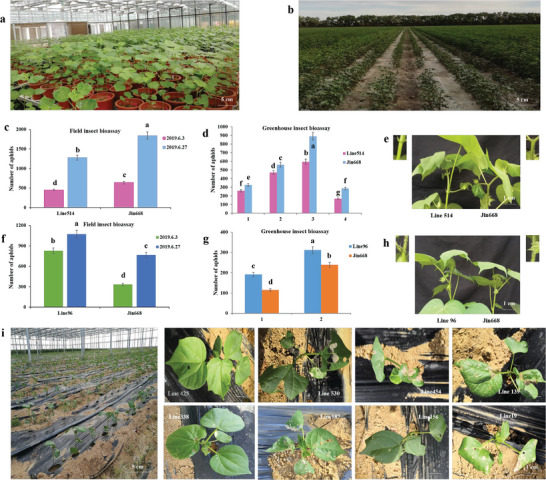
Screening for resistant and sensitive materials by insect bioassay. a) Insect bioassays of the mutants grown in the field of Xinjiang city. b) Insect bioassay of the mutants grown in the greenhouse in Wuhan city with aphid infestation. c–h) Aphid population size in the insect‐resistant line 514 and insect‐sensitive line 96 from greenhouse and field tests. i) Insect bioassay on cotton mutants with chewing pests in the greenhouse in Hainan city.

In order to screen potential cotton host insect resistance genes, an insect bioassay was carried out to study the response of the edited lines to insect infestation. A total of 200 independent T1 lines targeting 133 genes were used for insect bioassays under greenhouse and field conditions. First, plants of T1 generation were tested with aphids in the greenhouse, while the T2 generation was verified under field condition (**Figure** **3**a,b). Ten lines significantly responded (*p* ≤ 0.05) to aphid infestation, in both greenhouse and field experiments. Among them, two lines showed higher resistance to aphid infestation with a significantly lower number of aphids than that in the control plants (Figure 3c–e, Figure S6, Supporting Information). On the other hand, the number of aphids on the other 8 lines was significantly higher than that of the control plants, representing increased susceptibility to aphids (Figure 3f–h, Figure S6, Supporting Information).

The response of these 200 cotton lines was also examined with chewing pests (cotton bollworm, cabbage worm and leaf roller) in the field. Based on the assessment of the damaged leaves, 9 lines exhibited varying damage levels compared to the control plants. Among them, lines 338 and 425 showed an extreme anti‐insect phenotype with minor damage, while the other 7 lines showed an extreme insect‐sensitive phenotype (Figure 3i). These results indicate that more than 10% of the 133 tested mutants with targeted edited genes exhibited altered responses to insect infestation, which were found to be involved in plant defense to insect infestation based on gene annotation analysis (Figure S1, Supporting Information). The editing profiles of all lines showing altered responses to insect infestation were examined, and the results showed that these lines exhibited effective genome editing with a high genome editing frequency (**Table** **3**). In a nutshell, the above results suggest that the pooled mutant library targeting a specific trait (insect resistance in this case) is a promising strategy to identify and screen a wide range of functional genes.

**Table 3:** Responsive genes from the mutant library related to cotton insect resistance.

Mutant line no.	Gene ID	Aphid resistance	Chewing pests resistance	Type of mutation at the target site	Editing frequency
**Line 514**	Gh__A05G1815	R	NR	24/1, −42, −16/1, +3‐11/2	100%
**Line 96**	Gh__A04G0555	S	NR	−1	100%
**Line 561**	Gh_A05G1023	R	NR	+33, −1, +1, −7, −9/3	62.27%
**Line 539**	Gh_A03G0298	S	NR	−1,/1, −3	58.01%
**Line 172**	Gh_A10G1104	S	NR	+1, −1	100%
**Line 24**	Gh_A10G0762	S	NR	+1, −2	74.89%
**Line 293**	Gh_A06G0136	S	NR	−2, −17,/2	100%
**Line 139**	Gh__A03G1240	S	S	−5, −1	100%
**Line 144**	Gh__A03G1240	S	S	−3,/2, +1	100%
**Line 187**	Gh__A07G1493	NR	S	‐3, −1	34.39%
**Line 530**	Gh__A11G0142	NR	S	+1, −21	66.67%
**Line 338**	Gh__A11G0358	NR	R	‐1, −54	100%
**Line 156**	Gh__A03G0121	NR	S	‐1, −4/1, −2/2	100%
**Line 454**	Gh__D03G1250	NR	S	−1	100%
**Line 425**	Gh__A12G1862	NR	R	−1, −2, −3, −33	100%
**Line 19**	Gh__D13G0941	NR	S	/1, −8	83.39%

R represents insect resistance, S represents insect sensitivity, NR represents no response and “+”, “−”, “/”represent nucleotide insertions, deletions and substitutions at the target site, respectively. The underlined gene is the gene ID of *GhMLP423*.

### 2.5 Evolution and Domestication of the GhMLP423 Locus in the 1623 Re‐Sequenced Cotton Population

From the previous analyses, lines 139 and 144 (designated as CR‐139 and CR‐144) harbored the same sgRNA targeting the *GhMLP423* gene that was successfully knocked‐out in both lines, the editing profiles of the two lines are shown in Figure S7, Supporting Information. Interestingly, both lines exhibited high vulnerability to both sap‐sucking and chewing insects, suggesting *GhMLP423* might play an essential role in cotton resistance to insect pests, which encouraged us to select them for further study. Therefore, we studied the evolution and domestication of the *GhMLP423* locus to understand its role in insect‐resistance during cotton evolution. The re‐sequencing data of 1623 cotton germplasms from our previous study^[^ ^29^ ^]^ were utilized, which included 256 *G. hirsutum* landraces (Ghlandraces), 438 elite *G. hirsutum* cultivars from the USA and other countries (GhImpUSO), and 929 elite *G. hirsutum* cultivars from China (GhImpCHN). First, single‐nucleotide polymorphism (SNP) variants adjacent to the *GhMLP423* locus (50 kb upstream and downstream) were used to classify cotton populations, and PCA results indicated that Ghlandraces had wider variability in population variation (Figure S8a, Supporting Information). The nucleotide diversity results also showed diverse evolutions of the Ghlandraces population at the *GhMLP423* locus (Figure S8b, Supporting Information). To further investigate the evolution of *GhMLP423* in the Ghlandraces population, we mapped the genotype of the *GhMLP423* locus and showed that *GhMLP423* contained SNPs at five loci, containing two in introns and three in the 3′ UTR, and analysis of haplotypes in the Ghlandraces population revealed that the main SNP locus was G to A at intron, accounting for 60% (Figure S8c, Supporting Information). Moreover, the RNA‐seq data showed that the transcription level of *GhMLP423* was significantly higher in the Ghlandraces population than the GhImpCHN population (Figure S8d, Supporting Information). Therefore, we hypothesized that the promoter region of *GhMLP423* might have important SNP variants. Results showed that there were 13 SNPs variants in the Ghlandraces population of *GhMLP423* promoter, of which the 99 786 545 loci was located in the TATA‐box(−30) on the promoter that affected *GhMLP423* expression (Figure S8e, Supporting Information). Generally, the Ghlandraces are generally insect‐resistant, indicating that *GhMLP423* was selected and retained in insect‐resistance processes during cotton evolution.

### 2.6 Identification of a Broad‐Spectrum Insect Resistance GhMLP423 Gene

**Figure 4:**
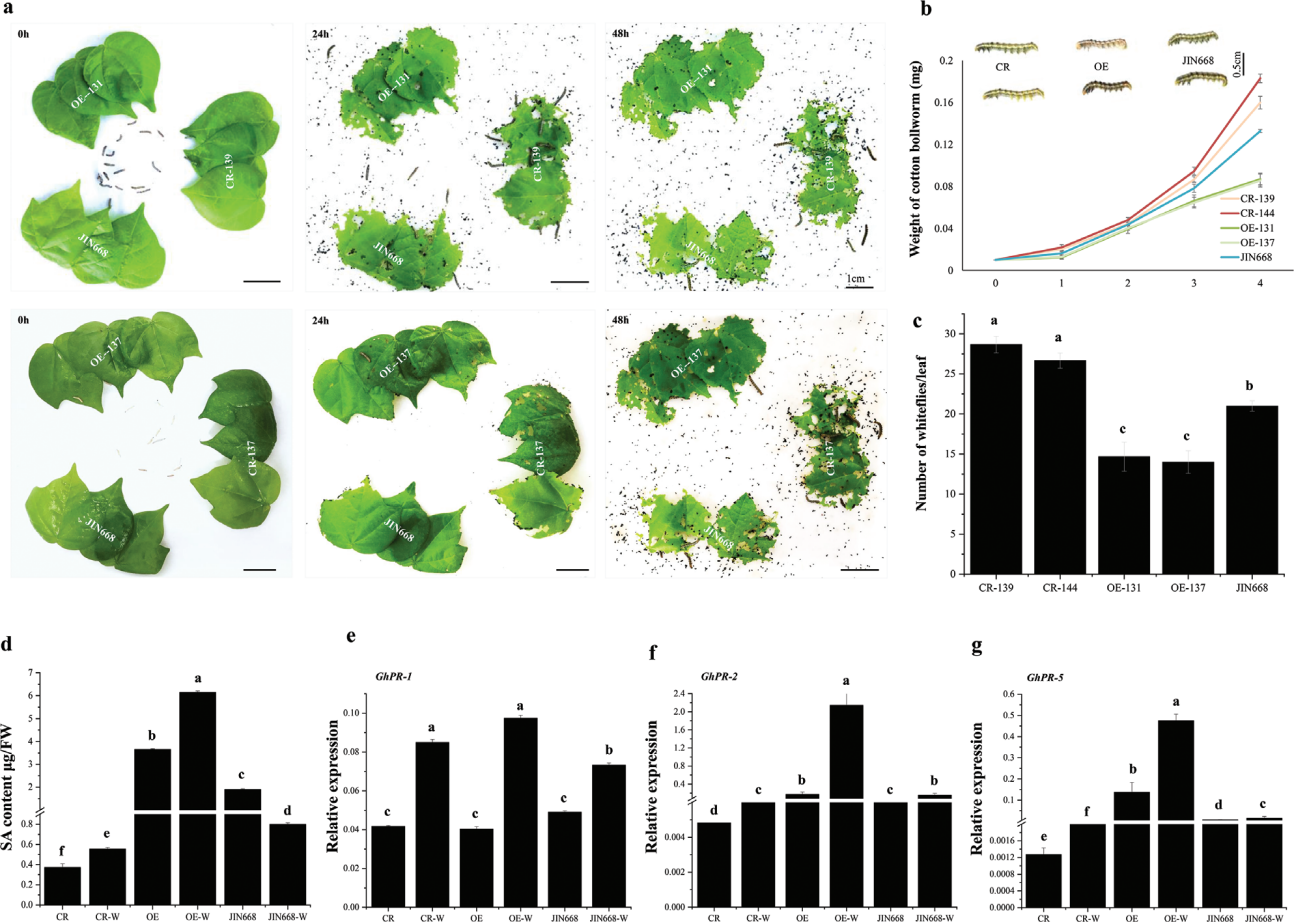
Identification of *GhMLP423* mutants. a) Cotton bollworm bioassay for *GhMLP423* knockout plants (CR), *GhMLP423* overexpressing plants (OE) and JIN668 (control plants). b) The weight of cotton bollworms larva (mg) fed on the leaves from each line was recorded at 48 h. Values are the means ± SD; *n* = 5. c) The number of whiteflies on the leaves from each plant line was recorded 7 days post infection. Values are the means ± SD; *n*  = 3. d) Total of SA content (µg FW^−1^) in non‐wounded CR, OE, JIN668 and wounded CR‐W, OE‐W and JIN668‐W seedlings. FW, Fresh weight. (e‐g) qRT‐PCR analysis of *PR‐1, PR‐2* and *PR‐5* genes in CR, OE and JIN668 plants. Values are the means ± SD; *n* = 3. Statistical analyses were performed using Student's *t*‐test *p* < 0.05.

Previous studies have reported that *MLP423* is a defense‐related and stress‐responsive gene.^[^ ^30^ ^]^ However, its regulatory mechanism in plant resistance to herbivores is largely unknown. Therefore, the role of this gene was studied in depth. In addition to the CRISPR/Cas9 knock‐out lines described above, transgenic cotton plants overexpressing *GhMLP423* gene were also generated, and two lines designated as OE‐131 and OE‐137 were selected for subsequent experiments. To study the role of *GhMLP423* in plant response to pest infestation, an insect bioassay was performed for *GhMLP423* mutant plants and compared to JIN668 plants (as control) with one chewing pest species (cotton bollworm) and one sap‐sacking insect species (whiteflies). The CR leaves were more severely damaged by cotton bollworm larvae (**Figure** **4**a), which showed a higher developed weight compared to JIN668 (0.17 mg vs 0.11 mg, respectively). While OE leaves were slightly damaged, this was reflected in the lower larvae weight (0.08 mg) (Figure 4b). For whitefly bioassay, CR plants were more susceptible to whitefly infestation than JIN668, whereas OE plants exhibited higher resistance to whitefly infestation. Notably, the number of whiteflies on CR plants was two times higher than that of OE (Figure 4c, Figure S9a, Supporting Information). These results indicate that *GhMLP423* is a positive regulator for cotton resistance to insect pests.

### 2.7 GhMLP423 Activates SAR

The plant SAR is characterized by the accumulation of SA and the coordinated activation of *PR* genes.^[^ ^31^ ^]^ To study the role of *GhMLP423* in SAR induction, SA content was tested in *GhMLP423* mutants and JIN668 plants after wounding treatment. All tested plants exhibited an increase in SA content in response to wounding (Figure 4d). However, OE plants accumulated the highest SA (0.96 and 1.61 mg ml^−1^) before and after treatment, while CR exhibited the least accumulation of SA (0.30 and 0.47 mg ml^−1^), respectively. Additionally, the transcript levels of three *PR* genes associated with insect resistance were detected in *GhMLP423* mutants and compared with JIN668 before and after wounding treatment. The transcription of *PR‐1* showed no significant differences in all the tested lines (Figure 4e). Whereas the transcription of *PR‐2* and *PR‐5* significantly increased in OE before and after wounding by 3.4 and 12.3 folds and 18.4 and 23.0 folds, respectively. However, the transcription of *PR‐2* and *PR‐5* was repressed in CR by 10.5 and 15.6, and 5.8 and 4.5 folds before and after wounding when compared to JIN668, respectively (Figure 4f,g). The changes in SA content and the expression of *PR‐2* and *PR‐5* are in the same trend with the changing expression of *GhMLP423* (Figure S9b, Supporting Information) which might induce SAR, that in turn retarded insect feeding.

### 2.8 *GhMLP423* Elicits [Ca^2+^]_cyt_ Wound‐Induced Signaling/Responses

**Figure 5:**
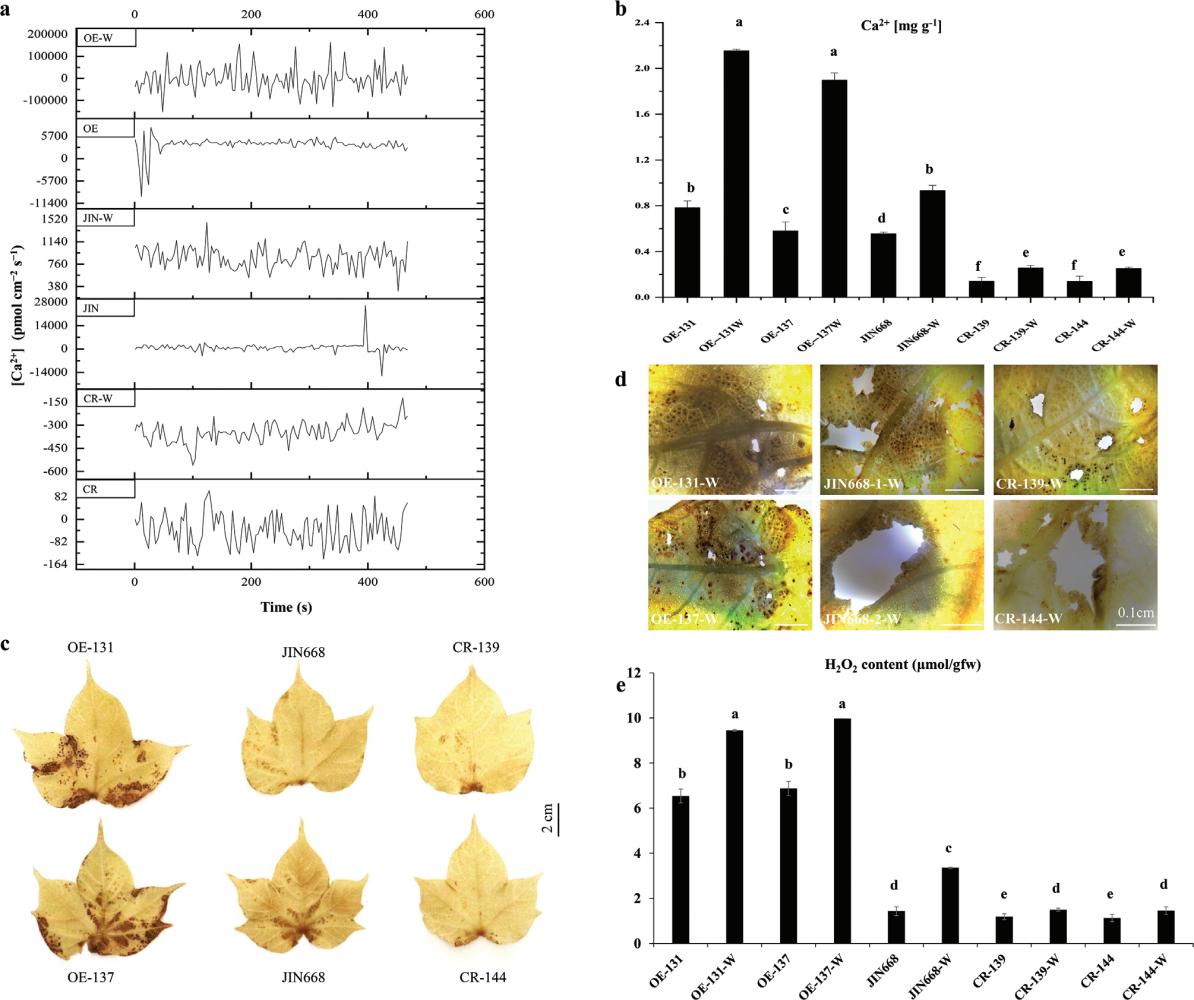
Ca^2+^ and ROS signaling of *GhMLP423* mutants in response to wounding. a) Measurement of Ca^2+^ flux in cotton leaves (pmol cm^−2^ s^−1^) of *GhMLP423* knockout (CR), *GhMLP423* overexpressing (OE) and JIN668 (control) seedlings the non‐invasive micro‐test technology (NMT) under normal and wounding (W) conditions. b) Content of Ca^2+^ ions (mg g^−1^) in leaves of CR, OE and JIN668 under normal and wounding (W) conditions. c) DAB Staining of CR, OE, and JIN668 leaves, the sporadic brown spots represent the accumulation of hydrogen peroxide (H_2_O_2_). d) DAB Staining of CR, OE, and JIN668 leaves, the sporadic brown spots represent the accumulation of hydrogen peroxide (H_2_O_2_) at the feeding sites of field pests. e) H_2_O_2_ content (µmol gfw^−1^) in CR, OE, and JIN668 leaves. gfw, green fresh weight. Values are the means ± SD; *n*  = 3. Statistical analyses were performed using Student's *t*‐test *p* < 0.05.

Spatial propagation of Ca^2+^ flux is one of the earliest cellular wound‐induced responses in plants, followed by membrane depolarization and changes in [Ca^2+^]_cyt_ levels. The role of *GhMLP423* to initiate wound signaling recognition was studied via detecting [Ca^2+^]_cyt_ fluxes in CR and OE plants compared to JIN668. The [Ca^2+^]_cyt_ flux was measured using non‐invasive micro‐test technology, and results showed that the average [Ca^2+^]_cyt_ flux induced by wounding was significantly higher in overexpressing lines than that of JIN668, while knockout plants exhibited poor wounding‐induced [Ca^2+^]_cyt_ flux compared to JIN668 plants (**Figure** **5**a). To measure the intracellular [Ca^2+^]_cyt_, a calcium colorimetric assay was used to detect Ca^2+^ ion content in the studied lines before and after wounding. Results showed a significantly lower level of Ca^2+^ content in CR (0.14 and 0.25 mg g^−1^) compared to JIN668 (0.55 and 0.93 mg g^−1^), while OE revealed a significantly higher content of Ca^2+^ (0.68 and 2.02 mg g^−1^) before and after wounding, respectively. No significant changes were observed in Ca^2+^ content between OE and JIN668 plants before wounding. However, higher Ca^2+^ accumulation was detected in OE plants compared to JIN668 after wounding by 2.1 times (Figure 5b).

### 2.9 Modulation of [Ca^2+^]_cyt_ Signaling Affects ROS Production in *GhMLP423* Mutant Plants

Ca^2+^ and ROS signals are stimuli‐induced signals acting as secondary messengers in plant immune and wounding responses. Several evidences suggested a mutual interplay between Ca^2+^ and ROS signaling in promoting fine‐tuning cellular signaling networks. To examine whether changes in [Ca^2+^]_cyt_ fluxes affect ROS production, the level of ROS accumulation was examined. Leaves from 8‐week‐old plants of CR, OE, and JIN668 were stained with 3,3′‐diaminobenzidine tetrahydrochloride (DAB) to detect the accumulation of hydrogen peroxide (H_2_O_2_) in situ. DAB‐stained OE leaves were the darkest among the tasted lines (Figure 5c), indicating a higher accumulation of ROS, especially at the wounding sites (Figure 5d). In addition, H_2_O_2_ content was quantified, and the results were in agreement with staining observations. The H_2_O_2_ level increased in OE before and after wounding by 4.6 and 2.6 times than JIN668, whereas the H_2_O_2_ level in CR decreased by 1.2 and 2.2 times than JIN668, respectively (Figure 5e). Changes in ROS might be due to changes in [Ca^2+^]_cyt_ fluxes and both signaling pathways involved in *GhMLP423*‐induced immune system.

### 2.10 GhMLP423 Interacts with a Calcium ‐Binding Protein (GhEPS15)

**Figure 6:**
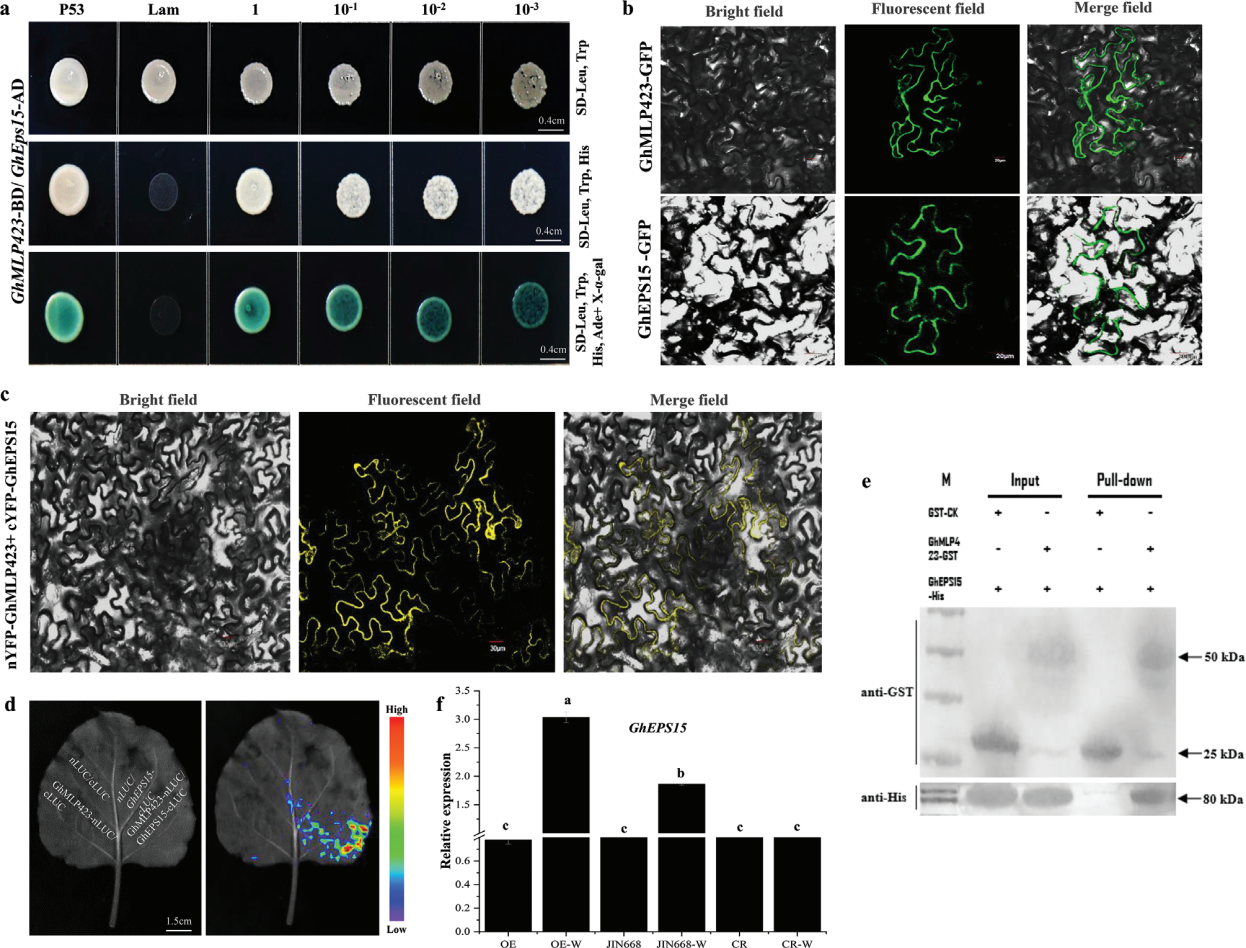
GhMLP423 physically interacts with GhEPS15 protein in vitro and vivo. a) Yeast two‐hybrid screening of the binding domain (GhMLP423‐BD) with the activation domain (GhEPS15‐AD). SC, Synthetic complete agar medium; SC‐ Leu, Trp: SC minus Leu and Trp; SC‐ Leu, Trp, His: SC minus Leu, Trp, and His; SC‐ Leu, Trp, His, Ade: SC minus Leu, Trp, His and Ade. b) Subcellular localization of GFP‐GhMLP423 and GFP‐GhEPS15 in epidermal cells of tobacco leaves. c) Bimolecular fluorescence complementation (BiFC) assay between GhMLP423‐nYFP and cYFP‐GhEPS15 in tobacco epidermal cells. d) Luciferase complementation imaging (LCI) assay of GhMLP423‐nLCI and cLCI‐GhEPS15 co‐expression in tobacco epidermal cells. e) Pull‐down assay, GhMLP423‐GST, but not GST‐CK, can be co‐immunoprecipitated with GhEPS15‐His. f) qRT‐PCR analysis of GhMLP423 gene in CR, OE and JIN668 plants. Statistical analyses were performed using Student's *t*‐test *p* < 0.05.

To understand how *MLP423* functions in plant defense, co‐expressed genes were screened by a yeast two‐hybrid (Y2H) assay using a cotton endogenous wound‐induced protein library. In the initial screening, the GhMLP423‐BD (BD: GAL4 DNA‐binding domain) protein showed positive interaction with two proteins, uncharacterized GPI‐anchored protein and uncharacterized calcium‐binding protein, as AD: activation domains (Figure S10, Supporting Information). Due to the changing calcium flux observed in *GhMLP423* mutants and the direct involvement of Ca^2+^ in plant defense against herbivores, we found that the uncharacterized calcium‐binding protein is an interesting candidate to select for further study. This protein is a homologue to the human epidermal growth factor receptor substrate 15 (EPS15) with a calcium‐binding EF hand‐containing protein domain. Therefore, the interaction between GhMLP423‐BD and GhEPS15‐AD was further confirmed by a point‐to‐point assay, which assured a positive interaction (**Figure** **6**a). The EPS receptor acquires extracellular ligands and transduces signals to the intracellular receptors via the cell membrane.^[^ ^32^ ^]^ Thus, the subcellular localization of GhMLP423‐GFP and GhEPS15‐GFP was detected, and results displayed that GhMLP423 is localized in the nucleus and cell membrane, while GhEPS15 is localized in the cell membrane (Figure 6b). Then, the physical interaction between GhMLP423 and GhEPS15 was further detected by the bimolecular fluorescence complementation (BiFC) assay. A yellow florescent signal was detected in the cell membrane using confocal microscopy when GhMLP423‐nYFP (N terminus of yellow fluorescence protein, nYFP) and cYFP‐GhEPS15 (C terminus of yellow fluorescence protein, cYFP) were co‐expressed in tobacco leaf epidermal cells (Figure 6c). Furthermore, a luciferase complementary imaging (LCI) assay was also conducted to verify this interaction by fusing the nLCI (N terminus of luciferase) to GhMLP423 and the cLCI (C terminus of luciferase) to GhEPS15. A positive co‐expression of GhMLP423‐nLCI and cLCI‐GhEPS15 proteins was observed in the epidermal cells of tobacco leaf (Figure 6d). Furthermore, a prokaryotic expression system was used to induce GhMLP423‐GST and GhEPS15‐His proteins for a pull‐down assay and results showed that GhMLP423‐GST could be co‐precipitated with GhEPS15‐His (Figure 6e). These results indicate that GhMLP423 and GhEPS15 co‐localize and directly interact at the cell membrane, which might open new insights to understand how *MLP423* is involved in plant immunity system. To further study the genetic interaction between *GhMLP423* and *GhEPS15*, the expression pattern of *GhEPS15* was examined in *GhMLP423* mutant plants. The transcription level of *GhEPS15* was not significantly changed in the mutant plants when compared to JIN668. However, the transcription level of *GhEPS15* was upregulated in JIN668 and OE plants in response to wounding treatment, while no significant change was observed in CR plants (Figure 6f). This upregulation was greater in OE plants than JIN668, indicating that *GhMLP423* and *GhEPS15* might synergistically work together to promote plant defense under pest attack.

### 2.11 GhEPS15 Positively Regulates Plant Defense Signaling

**Figure 7:**
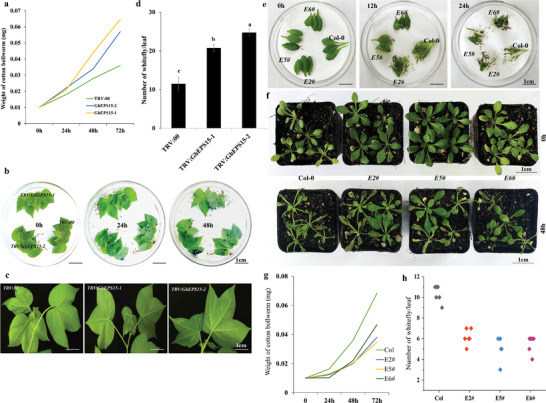
*GhEPS15* positively regulates plant resistance to insect infestation. a) The weight of cotton bollworm larva (mg) fed on the *TRV:GhEPS15* silenced, *TRV:GhEPS15*‐1, and *TRV:GhEPS15*‐2, and *TRV*:00 leaves was recorded at different time points. Values are the means ± SD; *n* = 5. b) Cotton bollworm bioassay for *TRV:GhEPS15*‐1, *TRV:GhEPS15*‐2 and *TRV*:00 leaves. c) Whiteflies colonization on the *TRV:GhEPS15* silenced and *TRV*:00 leaves. d) The number of whiteflies on the leaves from each silenced line was recorded 7 days post infection. Values are the means ± SD; *n*  = 3. e,f) Choice and non‐choice cotton bollworm bioassay for *GhEPS15* overexpression *Arabidopsis* lines, respectively. g) The weight of cotton bollworm larva (mg) fed on the *GhEPS15* overexpression *Arabidopsis* plants was recorded at different time points. Values are the means ± SD; *n* = 3. h) Number of whiteflies on the leaves of *GhEPS15* overexpression *Arabidopsis* lines, *E2#*, *E5#*, and *E6#*, was recorded 7 days post‐infection. Values are the means ± SD; *n*  = 5.

To investigate whether changes in the transcription level of *GhEPS15* are associated with plant resistance to pest attack, virus‐induced gene silencing (VIGS) was conducted to down‐regulate the transcription of *GhEPS15* in cotton. Results of qRT‐PCR showed that the transcript of *GhEPS15* was successfully suppressed in *TRV:GhEPS15‐1* and *TRV:GhEPS15‐2* 14 days after *Agrobacterium* infiltration (Figure S11a, Supporting Information). In addition, the coding sequence of the *GhEPS15* gene was cloned into an expression vector under the control of cauliflower mosaic virus (CaMV) 35S promoter and introduced into wild‐type *Arabidopsis*. Six overexpression transgenic lines with different expression levels were obtained (Figure S11b, Supporting Information), and three lines (*E2#*, *E5#* and *E6#*) with the highest expression level were selected for further analyses (Figure S11c, Supporting Information). A cotton bollworm bioassay was performed on *TRV:GhEPS15* and overexpression transgenic lines to examine the feeding preference of the second‐instar larvae and compare it to *TRV:00* and Col, respectively. Leaves of *TRV:GhEPS15‐1* and *TRV:GhEPS15‐2* were more damaged by cotton bollworm larvae than those of *TRV:00* (**Figure** **7**a,b), while leaves of *E2#*, *E5#* and *E6#* were less consumed by cotton bollworm larvae than Col, resulting in significantly lower larvae weight than those fed on Col (Figure 7e,f). Further, a whitefly two‐choice assay was also performed, and a clear difference was observed in whitefly colonization between *TRV:GhEPS15* and *TRV:00* plants, in which whitefly colonization was higher in *TRV:GhEPS15* than *TRV:00* (Figure 7c,d). Interestingly, the assay revealed that whiteflies were less attracted to *GhEPS15* overexpression plants as well (Figure 7h). These observations indicate that *GhEPS15* might positively regulate plant resistance to insects. To understand the relationship between *GhMLP423* and *GhEPS15*, the relative expression level of *GhMLP423* was analyzed in the silenced *TRV:GhEPS15* before and after wounding. Results showed no significant differences in transcription level between *TRV:GhEPS15* and *TRV*:00 infested and non‐infested seedlings (Figure S11d, Supporting Information).

**Figure 8:**
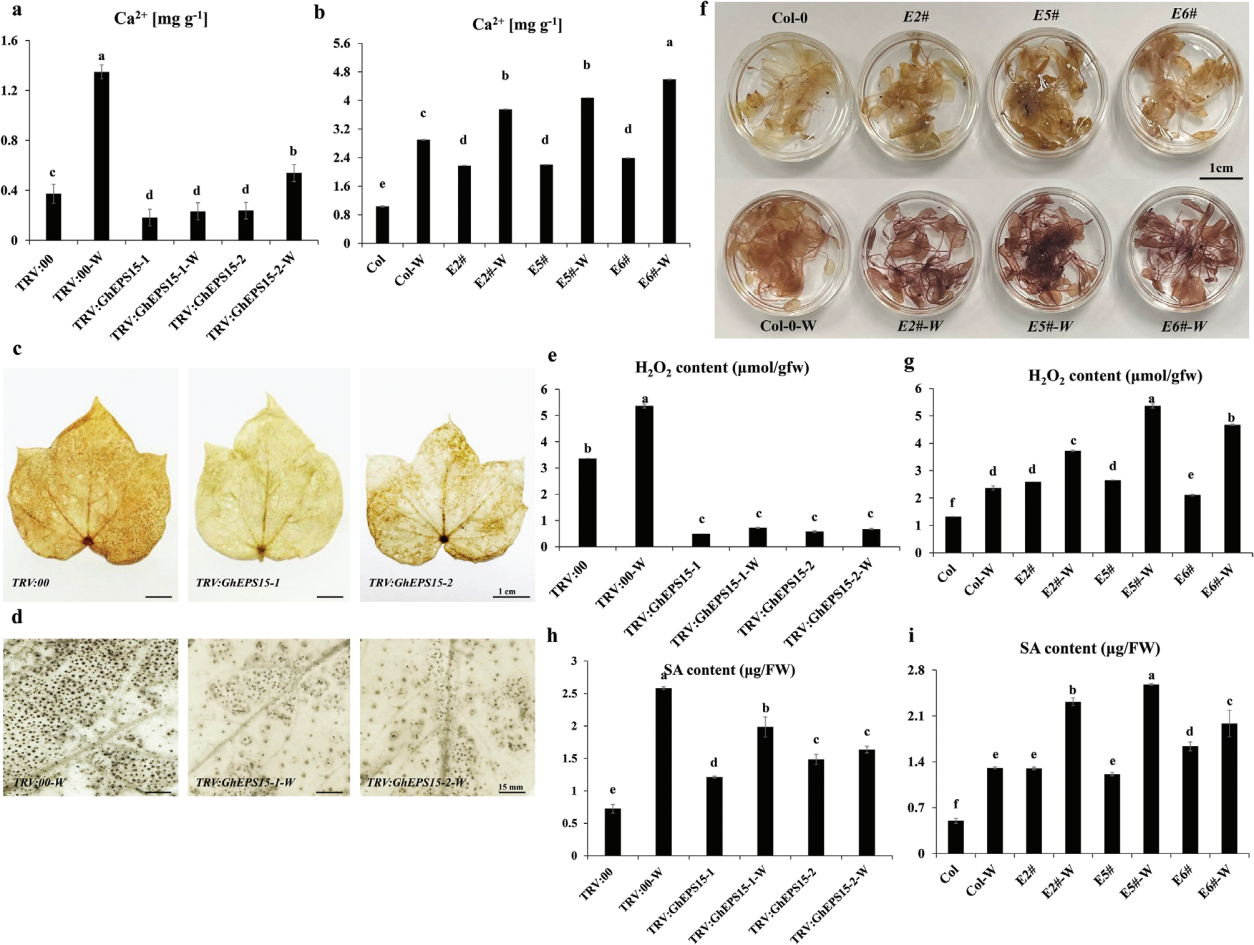
The role of *GhEPS15* on initiating Ca^2+^ and ROS signaling and SA responses. a,b) Content of Ca^2+^ ions (mg g^−1^) of *GhEPS15* silenced cotton and *GhEPS15* overexpression *Arabidopsis* under normal and wounding (W) conditions, respectively. c,d) DAB Staining of *TRV*:00 and *TRV:GhEPS15* non‐ and wounded leaves, respectively. Sporadic spots represent the accumulation of hydrogen peroxide (H_2_O_2_). e) H_2_O_2_ content (µmol gfw^−1^) in *TRV*:00 and *TRV:GhEPS15* non‐ and wounded leaves. gfw, green fresh weight. Values are the means ± SD; *n*  = 3. f) DAB Staining of *GhEPS15* overexpression *Arabidopsis* lines, *E2#*, *E5#* and *E6#*. The upper panel represents non‐wounded plants; and the lower panel represents wounded plants. g) H_2_O_2_ content (µmol gfw^−1^) in *E2#*, *E5#* and *E6#* non‐ and wounded plants compared to Col as a control. Values are the means ± SD; *n*  = 3. h,i) Total of SA content (µg FW^−1^) in *GhEPS15* silenced cotton and *GhEPS15* overexpression *Arabidopsis* non‐ and wounded leaves. FW, Fresh weight. Values are the means ± SD; *n*  = 3. Statistical analyses were performed using Student's *t*‐test *p* < 0.05.

To investigate how *GhEPS15* regulates plant resistance, the intracellular Ca^2+^ content was detected before and after wounding treatment. Results showed lower Ca^2+^ content in *TRV:GhEPS15‐1* and *TRV:GhEPS15‐2* than *TRV*:00 by 2.0 and 1.6 mg g^−1^ before and 5.8 and 2.5 mg g^−1^ after wounding, respectively (**Figure** **8**a). In contrast, *E2#*, *E5#* and *E6#* displayed significantly higher content of Ca^2+^ than that of Col before (2.1, 2.1 and 2.3 mg g^−1^) and after (1.3, 1.4, and 1.6 mg g^−1^) wounding, respectively (Figure 8b). Changes in Ca^2+^ content affected ROS accumulation in response to wounding treatment. Through DAB staining and chemical quantification, *TRV:GhEPS15‐1* and *TRV:GhEPS15‐2* leaves exhibited and accumulated lower H_2_O_2_ content than *TRV*:00 by 0.49 and 0.92 µmol g^−1^ before and 3.36 and 5.36 µmol g^−1^ after wounding, respectively (Figure 8c–e). The shortage of ROS in *TRV:GhEPS15* seedlings explains their sensitivity to insect infestation. Interestingly, overexpression of *GhEPS15* resulted in higher ROS production than Col, in which ROS was greatly elevated after wounding treatment (Figure 8f,g).

To determine whether *GhEPS15* contributes to plant SAR induction, the SAR indicators, SA, and *PR* genes were analyzed as well. Results showed that *GhEPS15* positively regulated SA accumulation, especially after infestation, as *TRV:GhEPS15‐1* and *TRV:GhEPS15‐2* seedlings exhibited lower SA accumulation after infestation by 1.6 and 2.1 folds than *TRV*:00 (Figure 8h), along with an extremely downregulated transcript of *GhPR‐1, GhPR‐2*, and *GhPR‐5* (Figure S12a–c). While overexpression of *GhEPS15* resulted in a significantly increasing accumulation of SA content compared to Col only after wounding treatment (Figure 8i). The transcription of *AtPR‐2* and *AtPR‐5* showed a significant increase in *GhEPS15* overexpression lines than Col (Figure S12e,f, Supporting Information), but not *AtPR‐1* (Figure S12d, Supporting Information).

**Figure 9:**
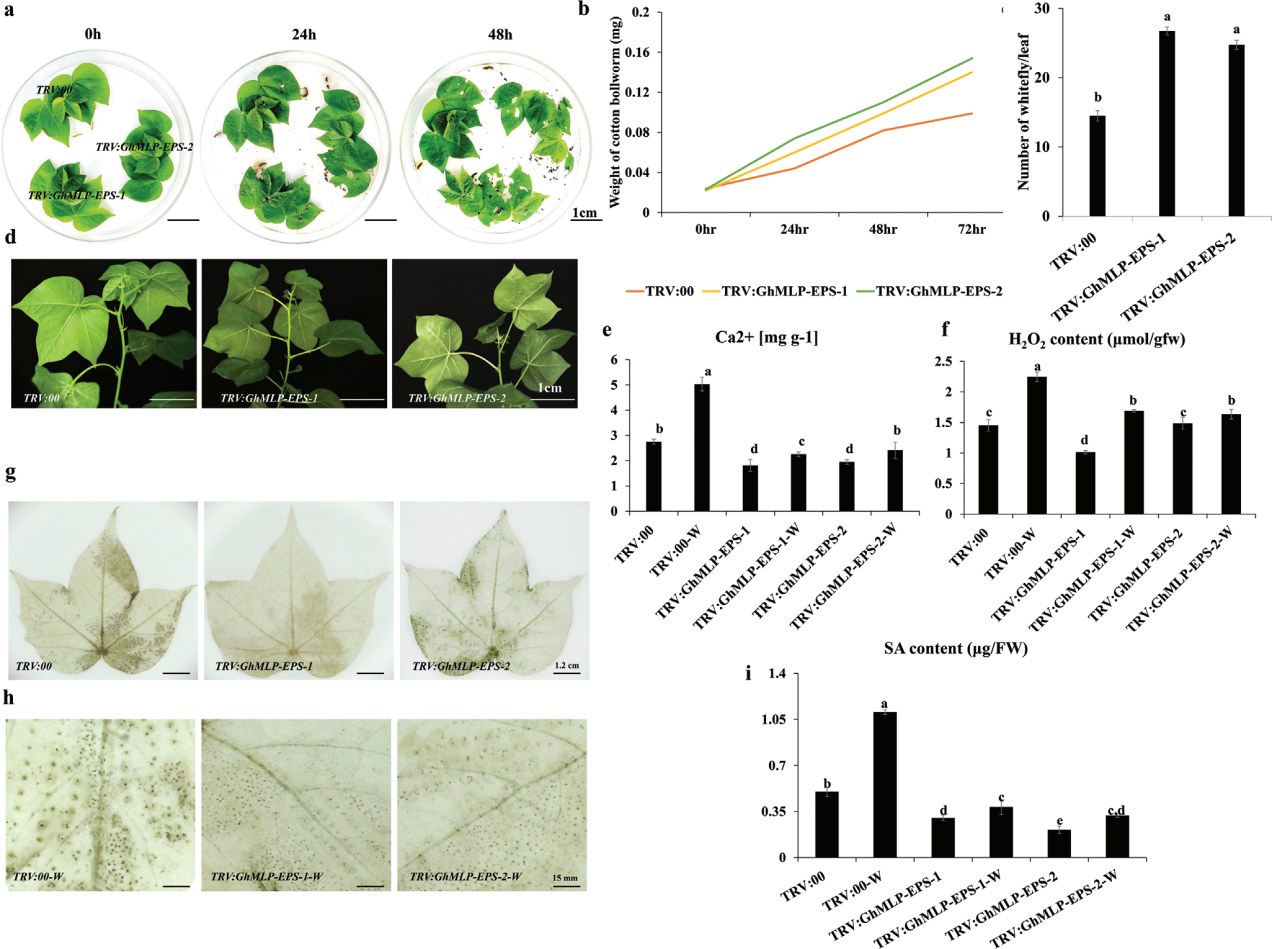
*GhMLP423* and *GhEPS15* cooperatively regulate Ca^2+^ and ROS signaling and SA responses. a) Feeding performance of cotton bollworm in co‐silenced *TRV:GhEPS15‐MLP* cotton leaves compared to *TRV*:00. b) The weight of cotton bollworm larva (mg) fed on the *TRV:GhEPS15‐MLP* co‐silenced and *TRV*:00 leaves was recorded at different time points. Values are the means ± SD; *n* = 5. c) The number of whiteflies on the leaves from each co‐silenced line was recorded 7 days post infection. Values are the means ± SD; *n*  = 3. d) Whiteflies colonization on the *TRV:GhEPS15‐MLP* co‐silenced and *TRV*:00 leaves. e) Content of Ca^2+^ ions (mg g^−1^) of *TRV:GhEPS15‐MLP* co‐silenced and *TRV*:00 leaves under normal and wounding (W) conditions. Values are the means ± SD; *n*  = 3. f) H_2_O_2_ content (µmol gfw^−1^) in *TRV*:00 and *TRV:GhEPS15* non‐ and wounded leaves. gfw, green fresh weight. Values are the means ± SD; *n*  = 3. g,h) DAB Staining of *TRV:GhEPS15‐MLP* co‐silenced and *TRV*:00 non‐ and wounded leaves, respectively. Sporadic spots represent the accumulation of hydrogen peroxide (H_2_O_2_). i) Total of SA content (µg/FW) in *TRV:GhEPS15‐MLP* co‐silenced and *TRV*:00 non‐ and wounded leaves. FW, Fresh weight. Values are the means ± SD; *n*  = 3. Statistical analyses were performed using Student's *t*‐test *p* < 0.05.

**Figure 10:**
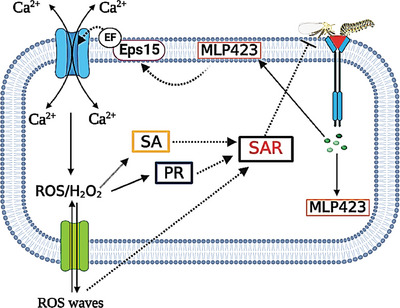
Schematic representation of insect‐wound induced cotton *MLP423* defense mechanism in cotton. Wound mediated by Insect induces the expression *MLP423* that activates SAR by the following mechanism: *MLP423* interacts with cotton *EPS15* (a calcium‐binding protein containing an EF‐hand motif) at the cell membrane that regulates wound‐induced Ca^2+^ flux as a second messenger. The induction of Ca^2+^ flux triggers reactive oxidative species (ROS) accumulation in the form of hydrogen peroxide (H_2_O_2_) and initiate cell‐to‐cell communication to activate SAR including the increasing levels of salicylic acid (SA) accompanied with the coordinated activation of (*PR*) genes.^[^ ^61^ ^]^ *MLP423*, major latex‐like protein 423; *EPS15*, epidermal growth factor substrate 15.

Finally, to further understand the regulatory network between the *GhMLP423* and *GhEPS15* genes, VIGS constructs for each gene were mixed equally to generate *TRV:GhMLP‐EPS‐1* and *TRV:GhMLP‐EPS‐2* co‐silenced cotton plants following the previously described method.^[^ ^33^ ^]^ Results of qRT‐PCR showed that both genes were successfully silenced in cotton plants 14 days after *Agrobacterium* infiltration (Figure S12g,h, Supporting Information). The phenotype of *TRV:GhMLP‐EPS‐1* and *TRV:GhMLP‐EPS‐2* was examined under cotton bollworm and whitefly infestation. Results showed that *TRV:GhMLP‐EPS‐1* and *TRV:GhMLP‐EPS‐2* were more susceptible to cotton bollworm feeding (**Figure** **9**a,b) and whitefly infestation (Figure 9c,d). This susceptibility was a result of the low Ca^2+^ content (Figure 9e), ROS accumulation (Figure 9f–h), and the SAR indicators of SA (Figure 9i), *GhPR‐1* (Figure S12i, Supporting Information), *GhPR‐2* (Figure S12j, Supporting Information) and *GhPR‐5* (Figure S12k, Supporting Information). Taken together, our results indicated that *GhMLP423* is a wound‐responsive gene that, together with *GhEPS15*, plays a positive role in regulating SAR in plant. *GhMLP423* induces the expression of *GhEPS15*, which in turn triggers wound‐induced Ca^2+^ fluxes in cotton plants. The activation of Ca^2+^ fluxes acts as a second messenger and induces ROS accumulation, which subsequently elicits the production of SA and PR proteins and activates the SAR to mediate insect resistance (**Figure** **10**).

## 3 Discussion

### 3.1 The Establishment of a CRISPR/Cas9 Mutant Library Targeting Specific Traits is Feasible for Plant Species with Complex Genomes

Mutagenesis plays an important role in functional genomics.^[^ ^34^ ^]^ The CRISPR/Cas9 system can rapidly and efficiently generate diverse mutant alleles in plants, including polyploid species.^[^ ^35^ ^]^ The conventional CRISPR/Cas9 system usually targets one or a few genes in one transformation step.^[^ ^28^ ^]^ Due to its capability for high throughput, the CRISPR/Cas9 system has been recruited to generate large‐scale mutagenesis libraries in different plant systems. In rice, genome‐wide mutant libraries covering thousands of genes have been constructed using array synthesized primers and pooled transformation to generate large‐scale of mutant populations, while the identity of sgRNAs in individual transgenic lines was characterized by barcoded PCR and next‐generation sequencing.^[^ ^36^ ^]^ In soybean, a pooled CRISPR/Cas9 plasmid library was constructed and transformed to create a multiplex mutant population.^[^ ^37^ ^]^ In addition, using the advantage of CRISPR/Cas9‐mediated large‐scale mutation, targeting genes related to a certain trait allows fast identification of elite genes to improve targeted traits. In cotton, a large‐scale mutant library targeting genes related to male sterility was generated via a pooled‐sgRNA assembly.^[^ ^28^ ^]^ From this point of view, we demonstrated the utility of the high‐throughput approach to generate an insect‐resistant‐related mutant library in cotton. The methodology described here uses the CRISPR/Cas9 system to construct a library of pooled plasmids (vectors) using hundreds of sgRNAs targeting hundreds of target sites across the cotton genome in one time and rapidly generates a saturated mutant library for insect resistance improvement (Figure 1). Overall, we demonstrate that building a CRISPR/Cas9 mutant library for large‐scale genes is feasible for polyploid plants with large genomes that can achieve a highly efficient target editing rate (Figure 2) and allow the analysis of complex agronomic traits.

### 3.2 Heritability of T‐DNA and Mutations in T1 Progeny

In cotton, the CRISPR/Cas9 system is still in its infancy, and the genetic basis of editing transmission to the progeny is largely unknown and is not following the classical genetic laws.^[^ ^38^ ^]^ Despite the vast majority of progenies inheriting the same mutation types as T0 parental plants, editing of T1 generations was improved in some lines exhibiting a low editing rate in T0 (Figure S5, Supporting Information). This phenomenon confirms that the CRISPR/Cas9 system can continuously edit genes across generations. The improved editing rate in the T1 generation indicates that T0 plants (containing the T‐DNA insertion of the CRISPR/Cas9 unit) can still be used to generate desired phenotypes in the offspring when the editing rate is low. In addition, new editing types with complicated genetic rules occurred in the offspring plants (Figure S5c, Supporting Information). Similar unusual transmission phenomena have been reported in other plant species such as soybean and maize,^[^ ^39^ ^]^ which may be related to chimerism in genome editing or the retention of Cas9 activity across generations. On the other hand, due to the transmission capability of editing through generations, the CRISPR/Cas9 unit can be isolated in the segregating generations, leading to edited transgene‐free progenies. This allows the creation of “transgene‐free” genetically modified organisms (GMOs), which would facilitate the commercial release of genetically modified plants. Therefore, it is possible to isolate transgene‐free plants with edited genes and desirable new phenotypes, which would be significant to resolve the issue of the commercial release of genetically modified plants in some parts of the world.

### 3.3 Optimization and Prospect of Gene Editing Tools in Cotton

Even though the CRISPR/Cas9 vector *pRGEB32‐GhU6.7* works well in cotton, it still needs further optimization that will be address blew. (i) The selection of CRISPR/Cas9 target sites requires NGG Protospacer Adjacent Motif (PAM), which makes it impossible to screen suitable target sites in genomic regions lacking the complementary sequence. We have recently established highly efficient CRISPR‐LbCpf1 and CRISPR‐Cas12b systems that have expanded the scope of genomic editing in cotton.^[^ ^40^ ^]^ Multiple Cas9 variants can also be further developed, such as xCas9 and Cas9 NG.^[^ ^26^ ^]^ (ii) The incidence of chimeras is another major challenge in plant genome editing, whereby the genome contains mixtures of edited and wild‐type DNA sequences in different cells.^[^ ^41^ ^]^ This phenomenon likely arises during the process of *Agrobacterium*‐mediated plant transformation and regeneration stages. If editing took place before the first cell division, homozygous edited plants could be obtained; nonetheless, delayed editing may lead to an increased frequency of chimerism and complex editing events.^[^ ^42^ ^]^ (iii) Several systems have been studied and applied to improve editing efficiency, including the use of an egg cell‐specific promoter, a meristem‐enriched YAO promoter‐driven system, and most recently, the replicon (LIR‐Donor‐SIR‐Rep‐LIR) of the bean yellow dwarf virus (BeYDV) to drive the transcription of sgRNA units in cotton.^[^ ^43^ ^]^ (iv) Temperature also affects the efficiency of the genome editing system. Cas9 is more active in *Arabidopsis* and Citrus at 37 than at 22 °C,^[^ ^44^ ^]^ while the CRISPR‐Cpf1 system also showed temperature‐sensitivity in plants.^[^ ^45^ ^]^ Since cotton is a thermophilic crop, it is grown at a quite high temperature that can reach up to 30 °C during tissue culture, with potential benefits of enhanced editing frequency during the regeneration phase. Besides, the temperature in most cotton cultivation regions in summer can reach 35°C, which will greatly increase the Cas9 enzyme activity. This may explain why the progeny of Cas9‐edited plants showed higher editing frequencies than the parent plants (Table 2).

### 3.4 Plant Responses to Pest Infestation Involve Complex and Overlapping Signaling Pathways

Insect pests seriously affect crop production, so it is of great importance to identify genes related to insect immunity. The molecular mechanisms governing the interaction between cotton and pests are still largely unknown. Plants have evolved complex defense networks, including highly complex signaling pathways, in response to abiotic and biotic stresses. After stress recognition, the immune system of plants triggers a variety of defense mechanisms that orchestrate transcriptional reprogramming through receptors/sensors, signal transduction cascades, hormone signaling, Ca^2+^ signaling, and ROS production.^[^ ^46^ ^]^ In this study, some mutant phenotypes were identified via insect bioassay under both greenhouse and field conditions (Figure 3). Interestingly, more than 10% of genes exhibited obvious insect‐resistance‐related phenotypes that need further verification (Table 3). As an example, in this study, *GhMLP423* loss‐of‐function mutants displaying high susceptibility to pest infestation were further studied in depth (Figure 3i).

### 3.5 The Domestication of *GhMLP423* Contributes to Ghlandraces Defense Against Insect Pests

Evolution and domestication are important for plant defense. The results showed that the expression level of *GhMLP423* in *G. hirsutum* landraces (Ghlandraces) was significantly higher than that in *G. hirsutum* cultivars from China (GhImpCHN), and there was an important mutation site in the promoter region (Figure S8, Supporting Information). In response to the fact that the exons of *GhMLP423* gene did not vary in all populations, we speculate that *GhMLP423* may be highly conserved in plants. We speculate that promoter variation may have influenced the expression level of *GhMLP423*, which in turn impacts the ability of plants to defend. In view of this, researchers can improve cotton defense traits in the cultivated species against this variant site (or other variants).

### 3.6 *GhMLP423* Triggers Systemic Wound Response by Activating Long‐Distance Signaling

To survive under environmental stresses, plants have evolved sophisticated signaling networks to coordinate their responses to the constantly changing environment. As signaling molecules, Ca^2+^, ROS, reactive nitrogen species, and others are the key cellular chemicals acting as “language codes” or so‐called second messengers to transfer the external information to the interior cell.^[^ ^47^ ^]^ In response to such signals, plants process this information into appropriate adaptive responses, including direct physiological responses as well as adjustment of gene expression, metabolic biosynthesis, and enzyme activity.^[^ ^15^ ^]^ Typically, a resistant plant is determined by how quickly and efficiently it can sense outer stimuli. Therefore, developing plants with superior immunity system is of great value for sustainable crop protection.

MLPs belonging to Bet v 1 protein family play critical roles in plant defense and stress responses. The Bet v 1 family comprises a large group of proteins, including cytokinin‐specific binding proteins (CSBPs), PR10 and major latex proteins (MLPs).^[^ ^20^ ^]^ The expression of different members of MLP was rapidly upregulated by mechanical stimulus,^[^ ^20^ ^]^ while it was downregulated after oxidative stress.^[^ ^48^ ^]^ The MLPs were also induced in response to pathogen infections. Although a number of studies have reported *MLPs* as responsive genes to pathogen invasion, the biological function of these proteins in plant signal transductions under pest attack is poorly understood. In this study, we identified the role of *GhMLP423* in cotton pest resistance. As shown in *CR* knockout mutants derived from our vector pooled library, loss‐of‐function of *GhMLP423* negatively influenced cotton resistance to two major pest insects, whiteflies and cotton bollworm, while overexpression of *GhMLP423* led to a developed resistance (Figure 4a–c, Figure S9a, Supporting Information). These results suggest that upland cotton's *MLP423* confers resistance against herbivory insects.

The *MLPs* enhance plant resistance through activating the SAR defense system. The *MLP‐PG1* from *Cucurbita pepo* enhanced disease resistance against fungal pathogens via the induction of *PR* genes, *PR‐2* and *PR‐5*.^[^ ^26^ ^]^ On the other hand, the growth of cotton leafworm larvae was retarded when fed with tobacco leaves expressing high levels of *PR‐1, PR‐2*, and *PR‐5* genes.^[^ ^49^ ^]^ In agreement with Fujita et al. findings,^[^ ^39^ ^]^ overexpression of *GhMLP423* in cotton induced the accumulation of SA and upregulated *PR‐2* and *PR‐5* genes but not *PR‐1* (Figure 4d–g), which most likely enhanced resistance to cotton bollworm, whiteflies and aphid infestation in *GhMLP423* overexpression plants due to their toxicity. However, the mechanisms underlying *MLP*‐mediated the induction of *PR* genes are still unknown. Therefore, the upstream defense lines in *GhMLP423* mutants were studied, which might involve in the systematic response of SA and *PR* genes.

The interaction between Ca^2+^ and ROS signals and the host of other signaling signatures is required for appropriate plant immune responses. The [Ca^2+^]cyt and ROS waves were originally proposed to interact in response to biotic and abiotic stresses. Having the ability to turn on plant systematic responses, *GhMLP423* promotes the dynamic elevation of [Ca^2+^]_cyt_ and ROS accumulation (Figure 5a–e) coupled with an increase in the expression of the *GhEPS15* gene in response to wounding (Figure 6f). The EPS15 is a calcium‐binding protein containing an EH‐domain that interacts with the EPS receptor to attach to certain ligands from the extracellular matrix (animal cell) and then transduces the signal into the inner membrane.^[^ ^32^ ^]^ The positive interaction between GhEPS15 and GhMLP423 proteins explains the changes in [Ca^2+^]_cyt_ levels in *GhMLP423* mutants and reveals a new and novel regulatory mechanism of *MLP423* (Figure 6a–e). This interaction is localized in the cell membrane, suggesting that both genes together initiate wound signaling recognition by activating Ca^2+^ signaling at the sensing tissues to promote plant defense system of SA and PR proteins. To ensure ROS production is a result of Ca^2+^ elevation, H_2_O_2_ production was detected in *TRV:GhEPS15* silenced cotton, *GhEPS15* overexpression *Arabidopsis*, and *TRV:GhEPS15‐MLP* co‐silenced cotton. Results supported our prospects showing that the decrease in [Ca^2+^]_cyt_ probably attenuates ROS accumulation, and as a result, influenced *TRV:GhEPS15* resistance or vice versa (Figures 8, 9). This can be elucidated by the fact that Ca^2+^ signals probably augment ROS flux through activating aquaporins with an EF‐hand motif.^[^ ^47^ ^]^ Unlike *GhMLP423, GhEPS15* expression is regulated in a *GhMLP423*‐dependent manner; this can be explained by the linear changes in the transcript level of *GhEPS15* in *GhMLP423* mutant plants in response to pest infestation. In contrast, no significant changes were obtained in the expression level of *GhMLP423* in the *TRV:GhEPS15* down‐regulated cotton before and after wounding (Figure S11d, Supporting Information) Accordingly, *GhMLP423* seems to positively regulate *GhEPS15* as it is accumulated in the nucleus, suggesting that *GhMLP423* might act as a transcriptional regulator. In the same manner, it's been suggested that *GhMLP28* functions as a transcriptional regulator of the *GhERF6* gene to enhance cotton resistance to *Verticillium* wilt disease.^[^ ^22^ ^]^

To sum up, this study proposes a critical line of evidence of the association of *GhMLP423* in plant defense by activating plant systematic responses via the systemically propagating waves of Ca^2+^‐induced ROS burst. Our results shed the light on new biological functions of *GhMLP423* and its association with Ca^2+^ and H_2_O_2_ signaling networks, which needs further clarification. Moreover, the human homologous *EPS15* gene, *GhEPS15*, was identified as an important candidate involved in plant defense responses and is required for *GhMLP423*‐mediated wound‐induced responses. To our knowledge, this is the first report tackles the role of *GhEPS15* in plant defense and its relationship with *GhMLP423* to mediate plant SAR defense (Figure 10). Finally, the detailed mechanism of how *GhEPS15* and [Ca^2+^]_cyt_ regulate ROS production needs further clarification.

## 4 Experimental Section

### sgRNA Design

In order to identify endogenous genes related to insect resistance in cotton, a large‐scale sgRNA library targeting 502 insect resistance‐related DEGs was selected. CRISPR‐P 2.0 software was used to design the sgRNA target sites for each gene, and *Gossypium hirsutum* (v1.1) genome was used as the reference sequence.^[^ ^50^ ^]^ Genome‐wide comparison screening was performed, and at least one gRNA with the highest scores was selected for each target gene. However, a small number of genes only have one ideal site to ensure specificity and reduce off‐target efficiency. All target selections followed the rule of the lowest possibility of off‐targets and the mismatch value of the target sequence was greater than 2 mismatches.

### GO Function Enrichment Analysis of Target Genes

Transcriptome data of cotton plants infected by sap‐sucking insects was derived from our previous report.^[^ ^51^ ^]^ Specifically, we use the oral secretions of *Helicoverpa armigera* and *Spodoptera litura* to apply on mechanically wounded cotton leaves to simulate wound‐responsive genes; only wounded leaves and wounded leaves treated with water were used for comparison. The most likely 502 DEGs related to insect resistance were identified and used as the input parameter (Table S1, Supporting Information). The Gene ontology (GO) data were used to annotate the function of these genes on the cotton reference genome. The software clusterProfiler was used for the enrichment analysis:http://bioconductor.org/packages/release/bioc/vignettes/clusterProfiler/inst/doc/clusterProfiler.html.

### Construction of a Pooled sgRNA Library

Vector used in this experiment was derived from the highly efficient plasmid vector *pRGEB32‐GhU6.7* developed by our lab for cotton genome editing.^[^ ^52^ ^]^ A total of 5 ng of *PRGEB32‐GhU6.7* plasmid was digested using BstBI‐HF for 6–8 h at 37 °C to ensure adequate digestion, and the digested mixture was purified. To ensure coverage and ease of operation, 40 primers were mixed in equal amounts to amplify the target sequences. The PCR products were directly ligated to CRISPR/Cas9 *pRGEB32‐GhU6.7* vector using ClonExpress II One Step Cloning Kit (Vazyme). Heat shock was performed to transform the constructed plasmid vector into *Escherichia coli*. After 15 h of growth, all *E. coli* strains were collected, and plasmids were extracted using TIANprep Mini Plasmid Kit (TIANGEN, Beijing, China). The concentration of each plasmid was measured spectrophotometrically, and all plasmids were mixed equally for *Agrobacterium tumefaciens* transformation. Two days later, all the *Agrobacterium* colonies were harvested by scraping and mixed into a pool for cotton transformation. All the steps are graphically illustrated in Figure 1.

### *Agrobacterium*‐Mediated Genetic Transformation

For cotton transformation, the cotton cultivar JIN668, which has high regeneration efficiency, was used for *Agrobacterium*‐mediated transformation, according to our previous publications^[^ ^53^ ^]^ with minor modifications (Figure S2, Supporting Information). Briefly, cotton seedlings were grown for 6 days in the dark, cut into 5–7 mm length, and then inoculated with the pooled *Agrobacterium* strains. Hypocotyls and *Agrobacterium* were incubated at 21°C in the dark for 48 h and then transferred to the callus induction medium for 2–3 months until somatic embryogenesis was initiated. After that, the embryonic callus was placed on a differentiation medium to regenerate plantlets, and finally, the regenerated plantlets were transferred to rooting medium for root formation. Genomic DNA was extracted from the young cotton leaves using a Plant Genome Extraction Kit (TIANGEN, Beijing, China).

For Arabidopsis transformation, the full‐length coding sequences of *GhEPS15* were amplified from the cDNA of *G. hirsutum*. The amplified cDNA fragments were subsequently constructed into the pGWB417 vector under the control of the 35S promoter for overexpression. The resulting constructs were genetically transformed into *Arabidopsis* Col‐0 using *agrobacterium*‐mediated floral dip method. The positive transgenic plants of T1 generation were screened out by kanamycin resistance and PCR genotyping. The highly expressed transgenic T1 lines were detected by RT‐PCR using the primers listed in Table S5, Supporting Information and pollinated for T2 generation, and used for further analyses.

### Barcode Design and High‐Throughput Sequencing to Detect Target Sequences

In combination with barcode strategies and high‐throughput sequencing analysis technology, the first step was to determine the target sequences of sgRNAs in regenerated cotton plants. To differentiate samples, 9‐nucleotide barcodes were added to the 5′ ends of the primers for amplification of fragments containing the sgRNA sequences. Using different combinations of forward and reverse primers, 44 primers could be used to detect 384 samples at one time. After PCR amplification, the PCR products were electrophoresed in a 1% agarose gel and detected under UV light. All PCR products were mixed in equal quantities into one sample and purified for high‐throughput sequencing. PCR‐free library construction was selected for next‐generation sequencing using the Nova‐seq platform. Unedited sequences were strictly compared, and the 20 bp target sequence was extracted from the perfectly matched sequence. The extracted 20 bp target sequences were filtered from the total sequences and compared with the corresponding sequence in each plant according to the barcode sequences.

### Genome Editing Detection

Primers for genome editing detection were designed corresponding to upstream and downstream regions of the sgRNA target sites. Illumina sequencing cannot sequence DNA fragments longer than 300 bp, so the length of PCR products was generally designed to be no more than 280 bp. Since the CRISPR‐Cas9 system can introduce considerable Indels at the target editing sites, the upstream and downstream primers should not be close to the target sites, and at least a 30 bp gap should be maintained when designing primers. In order to maintain consistent conditions for PCR amplification, the primer annealing temperature ranged between 58–60 °C. All PCR products were mixed and purified in equal amounts into one sample for high‐throughput sequencing.

### Insect Bioassays and Wounding Treatment

The first insect bioassay was conducted in the greenhouse at Huazhong Agricultural University, Wuhan City, Hubei, China. To maintain consistency, three random plants of each T1 line and a wild‐type (control) plant were planted in the same pot filled with nutrient soil. 200 T1 edited lines were randomly scattered in the greenhouse, with five replications. After two months of regular irrigation, weeding, and pest control, about 20 aphids were released onto each plant, and the total number of aphids in the whole plant was counted 1 week after the release of the insects. For a second insect bioassay in the greenhouse, the same 200 lines were tested at the China Cotton Research Institute (CCRI), located in San Ya city, Hai Nan province. At the seedling stage, the plants suffered from natural pests in the greenhouse. During the insect bioassay, leaf damage was recorded for all plants, and lines showing resistance or susceptibility to the insects were then selected according to the extent of the damage. In a 3rd insect bioassay experiment, the 200 edited lines were tested for resistance to aphids in field conditions in Alar city, where aphids cause serious yield losses. In order to avoid the uneven occurrence of aphids in large areas of the field, every five CRISPR/Cas9‐edited plants were planted in a row with one control plant, and 40 plants were planted in each experimental plot. Three plants were randomly selected from each plot, and the total aphid number on each plant was^[^ ^34^ ^]^ assessed twice: first from the entire 45‐day‐old cotton plants, and second from the top five leaves of 70‐day‐old cotton plants. Finally, an insect bioassay performed in a small scale was conducted in a growth chamber under controlled temperature and daylight hours (22–24 °C, 16 h). Plant leaves from the same position were harvested, placed together in a 15 mm Petri plate, and treated with pre‐starved first‐instar larvae for *Arabidopsis* and second‐instar larvae for cotton of cotton bollworm for 48 h. Wounding treatment was performed following the method described by.^[^ ^51^ ^]^ All samples subjected to artificial wounding were collected 60 minutes later from three individual cotton plants, flash‐frozen in liquid nitrogen, and stored at −80 °C. For the quantification of H_2_O_2_, wounded plants were kept under midlight for 48 h before sampling.

### qRT‐PCR and RT‐PCR

Total RNA was extracted from samples according to Zhu et al. (2005) and was then reverse‐transcribed to cDNA for gene expression analysis. The RT‐PCR procedure was as follows: one cycle of 5 min at 94°C as an initial denaturation step, followed by denaturation for 30 s at 94 °C, annealing for 30 s at 58 °C, extension for 30 s at 72 °C, and a final step at 72 °C for 6 min. qRT‐PCR was performed using the ABI Prism 7500 system (Applied Biosystems, Foster City, CA, USA). A 20‐µl reaction mixture containing diluted cDNA and Green Super‐mix was used for RT‐qPCR following the manufacturer's protocol. The procedure was as follows: 95°C for 1 min, 40 cycles of 95°C for 5 s, and 60 °C for 40 s.

### VIGS Procedure

The conserved region of *GhEPS15* was selected as a target for VIGS and then amplified from the leaf‐sample of cv. JIN668. PCR products were digested by two restriction endonucleases, BamHI and KpnI, and ligated to the *TRV* vector. Finally, the TRV vector was transformed into *Agrobacterium tumefaciens GV3101* through electroporation. *A. tumefaciens* harboring the *TRV* vector was infiltrated into two fully expanded cotyledons of 10‐day‐old seedlings. Seedlings were grown at 25 °C, 16/8 light/dark condition. For co‐silencing the two TRV constructs were equally mixed for *Agro*infiltration as previously described.^[^ ^33^ ^]^

### Y2H Assay

A cDNA library of cotton endogenous wounding‐induced proteins was used for Y2H screening using the Matchmaker Gold Yeast Two‐Hybrid System (Clontech, Cat. No. 630 489). The cDNA sequence of *GhMLP423* gene was fused to the GAL4 DNA‐binding domain in pGBKT7, tested for auto‐activation or toxicity by an X‐α‐Gal assay in yeast, and the GhMLP423 fusion protein was used as bait to identify interacting proteins. Only GhEPS15 protein was identified. Then the full‐length of GhEPS15 ORF was fused to the GAL4 DNA activation domain in PGADT7 for a protein‐protein detection assay.

### Sub‐cellular Localization, BiFC, and LCI Assays

To localize the GhMLP423 and GhEPS15 proteins, the cDNA sequences without stop codons were inserted into the C‐terminal GFP‐fusion expression vector PMDC84 (Curtis and Grossniklaus, 2003). Both vectors were introduced into *A. tumefaciens* strain *GV3101* for the transformation of 3‐week‐old tobacco leaves. Green fluorescent protein expression was observed 48 h after transformation under a confocal microscope (LeicaMicrosystems TCS SP2 AOBS, Germany). For the BiFC assay, the CDSs of GhMLP423 and GhEPS15 were cloned into pS1301nYFP and pS1301cYFP vectors, respectively. The pair of the two‐gene combination construct was transformed into *A. tumefaciens* strain GV3101, and then transiently expressed in *N. benthamiana* leaves by injection with needleless syringes. The fluorescence in the epidermal cells was observed 60 h later using the above confocal microscope. For the LCI assay, the full‐length CDS of *GhMLP423* and *GhEPS15* were cloned into JW771 and JW772 vectors. The vectors were transformed into *Agrobacterium GV1301* strain, and transient expression was performed in tobacco epidermal cells. LUC luminescence was examined using a cryogenically cooled CCD camera (Lumazome PyLoN 2048B).

### Pull‐Down Assay

For in vitro pull‐down assays, the CDS sequences *GhMLP423* and *GhEPS15* were cloned into the vectors pGEX‐4 T‐1 (Pharmacia) and PET‐28‐a (Novagen), respectively. The constructs GhMLP423‐GST and GhEPS15‐His were transformed into Escherichia coli BL21 (DE3). Empty GST and recombinant GhMLP423‐GST proteins were used to pull‐down the GhEPS15‐His. The pull‐down assay was performed as previously described.^[^ ^54^ ^]^

### Measurement of Extracellular Ca^2+^ Flux, DAB Staining, and H_2_O_2_ and SA Quantification

Leaves were used to detect Ca^2+^ flux using Non‐invasive Micro‐test Technology (NMT) as described previously.^[^ ^55^ ^]^ The Ca^2+^ content (mg·g^−1^) was measured using a calcium colorimetric assay kit (Beyotime Biotechnology Co., Ltd. Shanghai, China).

Leaves were incubated in 1 mg ml^−1^, pH 3.8, DAB‐HCl (Sigma‐Aldrich, USA) in the dark for 8 h. The cotyledons were then cleared by boiling in alcoholic lactophenol (95% ethanol: lactophenol, 2:1 v/v) for 20 min. The reddish color of the cotyledons was used as evidence of H2O2 and visualized using a Nikon D40 camera (Japan). The quantification of H_2_O_2_ was conducted using the test kit (G0112W) from Suzhou Grace Biotechnology Co., Ltd (Suzhou, China), following the manufacturer instructions. For SA extraction, plant tissues were ground into fine powder by liquid nitrogen, and 0.1 g was used from each sample following the method of.^[^ ^26^ ^]^ Samples were then analyzed using LC/MS.

### Evolution and Selection of the *GhMLP423* Locus in Resequencing Population

In this project, the re‐sequencing data and RNA‐seq data source is https://figshare.com/s/cb3c104782a1dcd90ab0.^[^ ^56^ ^]^ The VCFtools software (v0.1.16) was used to perform extraction of SNPs variants in samples, quality control, and calculates nucleotide diversity (π), –maf 0.01 –hwe 0.01.^[^ ^57^ ^]^ The plink software (v1.9) was used to perform format conversion.^[^ ^58^ ^]^ The GCTA software (v1.26.0) performs analytical PCA analysis –make‐grm –make‐grm‐alg 0.^[^ ^59^ ^]^ Gene structure was presented using the GSDS website (https://gsds.gao‐lab.org/) through the structure files of the genes. Gene promoter analysis was performed using the PlantCARE website.^[^ ^60^ ^]^ Haplotype analysis was obtained by analyzing SNPs variants using R scripts. Both pie charts and boxplots of gene expression were drawn by the R package ggplot2.

## Conflict of Interest

The authors declare no conflict of interest.

## Supporting information

Supporting Information

## Data Availability

The data that support the findings of this study are available in the supplementary material of this article.
