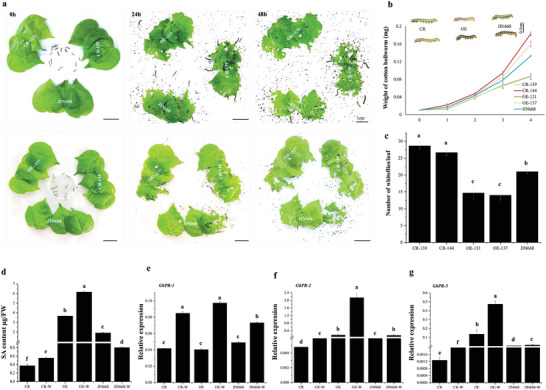# Correction to [Construction of Host Plant Insect‐Resistance Mutant Library by High‐Throughput CRISPR/Cas9 System and Identification of a Broad‐Spectrum Insect Resistance Gene]

**DOI:** 10.1002/advs.202404626

**Published:** 2024-05-24

[Sun L, Alariqi M, Wang Y, Wang Q, Xu Z, Zafar MN, Yang G, Jia R, Hussain A, Chen Y, Ding X, Zhou J, Wang G, Wang F, Li J, Zou J, Zhu X, Yu L, Sun Y, Liang S, Hui F, Chen L, Guo W, Wang Y, Zhu H, Lindsey K, Nie X, Zhang X, Jin S. Construction of Host Plant Insect‐Resistance Mutant Library by High‐Throughput CRISPR/Cas9 System and Identification of A Broad‐Spectrum Insect Resistance Gene. Adv Sci (Weinh). 2024 Jan;11(4):e2306157. https://doi.org/10.1002/advs.202306157]

[Upon careful review, we identified an unintentional typo in Figure 4a during manuscript proofing. The figure shows insect bioassay results for knockout and overexpression lines of the *GhMLP423* gene. Specifically, there was an error in including an incorrect image from the 1st biological replicate instead of the image from the 2nd biological replicate at the 48 h time point. To address this mistake, the incorrect image will be replaced with the right images representing one technical replicate from the 2nd biological replicate. Importantly, both replicates exhibited identical experimental outcomes, ensuring that this correction will not impact the overall validity or conclusion of the study.]