# Supplementary material for: Construction of Host Plant Insect‐Resistance Mutant Library by High‐Throughput CRISPR/Cas9 System and Identification of A Broad‐Spectrum Insect Resistance Gene
Source: Adv Sci (Weinh). 2023 Nov 30;11(4):2306157. doi: 10.1002/advs.202306157 (PMC10811493; doi:10.1002/advs.202306157)
Supplement: Supplementary file 1 — Supporting Information [file ADVS-11-2306157-s001.pdf]

ADVANCED SCIENCE
Open Access

Supporting Information

for *Adv. Sci.*, DOI 10.1002/advs.202306157

Construction of Host Plant Insect-Resistance Mutant Library by High-Throughput CRISPR/Cas9 System and Identification of A Broad-Spectrum Insect Resistance Gene

Lin Sun, Muna Alariqi, Yaxin Wang, Qiongqiong Wang, Zhongping Xu, Muhammad Naeem Zafar, Guangqin Yang, Ruoyu Jia, Amjad Hussain, Yilin Chen, Xiao Ding, Jiawei Zhou, Guanying Wang, Fuqiu Wang, Jianying Li, Jiawei Zou, Xiangqian Zhu, Lu Yu, Yiwen Sun, Sijia Liang, Fengjiao Hui, Luo Chen, Weifeng Guo, Yanqin Wang, Huaguo Zhu, Keith Lindsey, Xinhui Nie, Xianlong Zhang and Shuangxia Jin**

Additional files

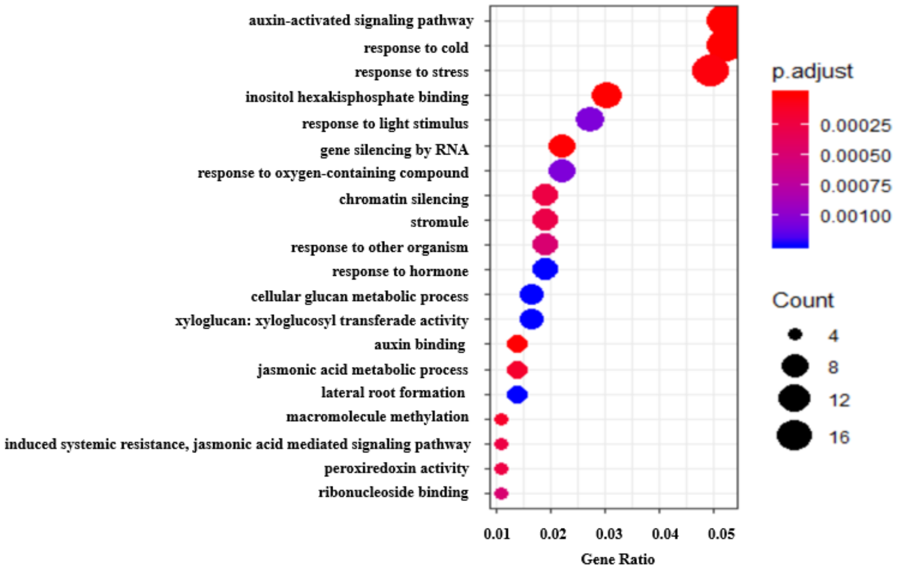

Figure S1

1

Figure S1. GO function enrichment analysis of 502 target genes. Pathways with higher confidence are shown in red with the adjust p-value presented. The dot size represents the number differences of GO enrichment.

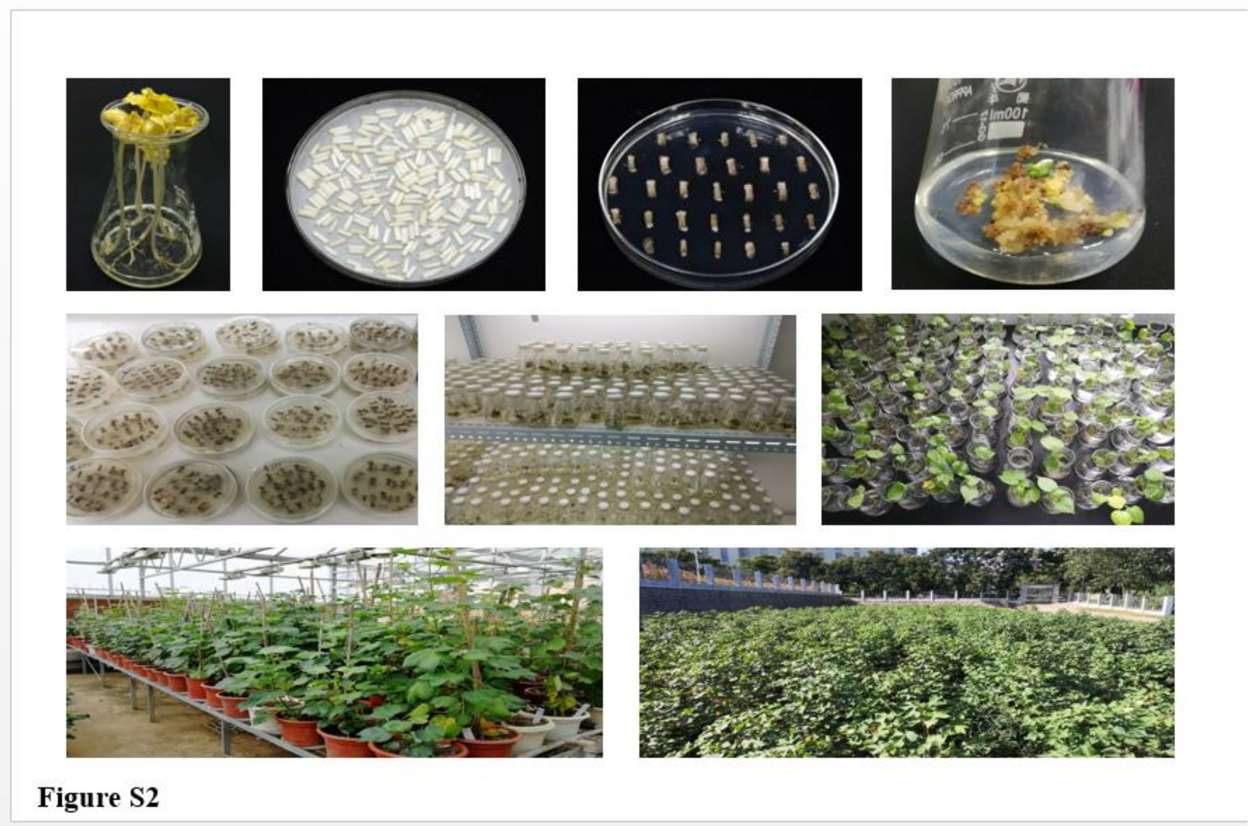

Figure S2. Genetic transformation and plant regeneration of cotton, greenhouse and field view of cotton mutants. (a) Seven-day ex-plants grown in the dark and used for transformation. (b-f) Callus induction and differentiation. (g) Plant regeneration. (h-i) Acclimatization of regenerated plants grown in growth room, greenhouse then field; respectively. Scale bars =1.1 cm.

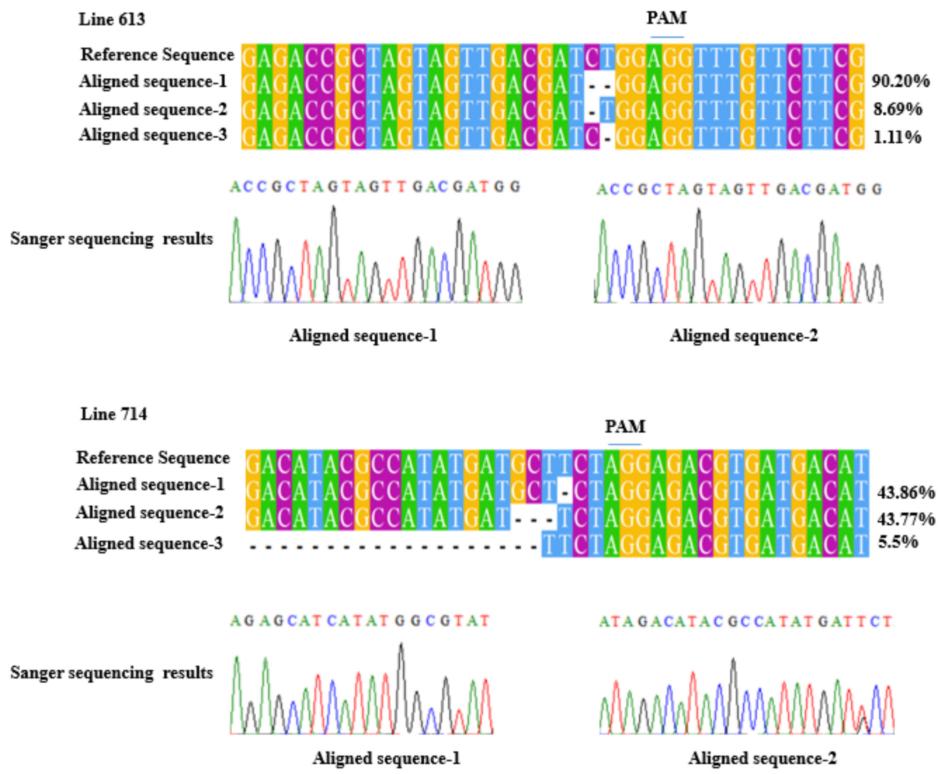

Figure S3

Figure S3. Sanger sequencing validated the high throughput sequencing data

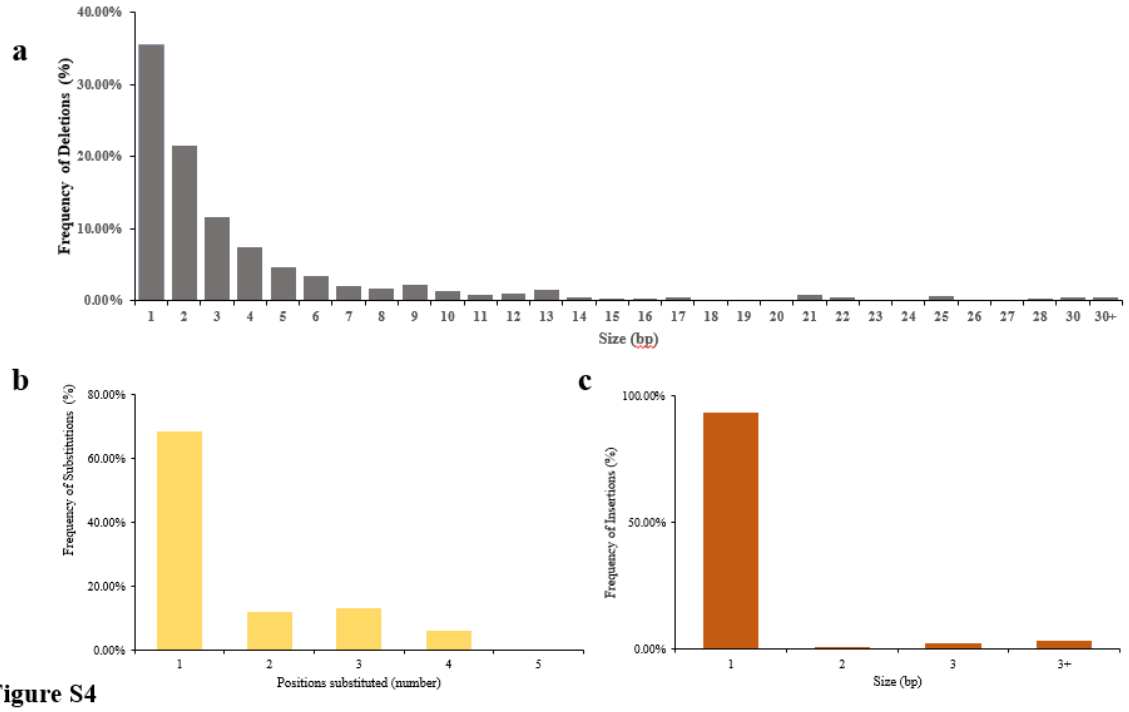

Figure S4

Figure S4: Detailed editing profiles of T0 plants in the mutant library. (a) Distribution of the length of deletions. (b) Different base numbers of substitutions. (c) Statistics of short fragment insertions with different lengths.

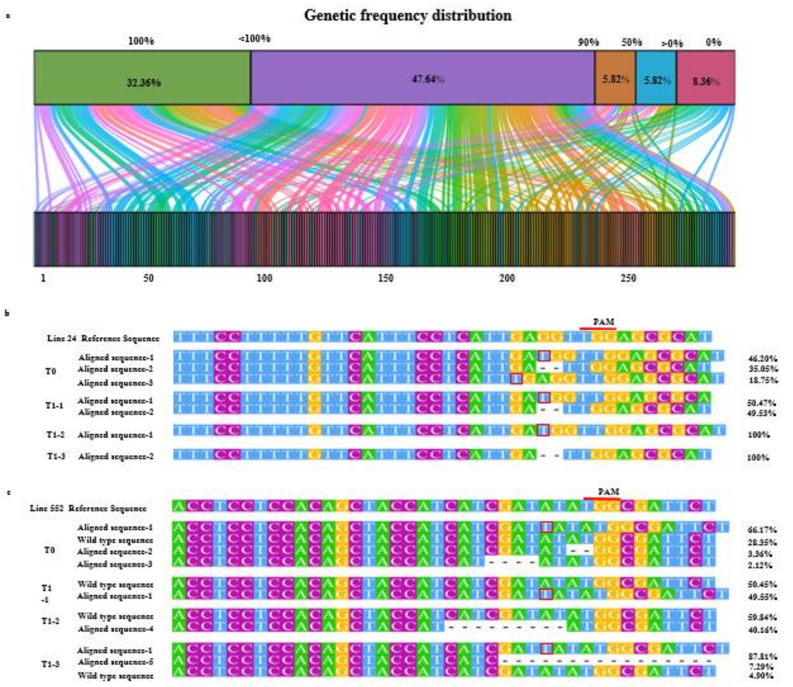

Figure S5

Figure S5. Inheritance of gene editing from T0 to T1 generation.
(a) Frequency of editing types in 276 T1 plants. One line represents a T1 plant and the same colored line comes from the same T0 plant. (b, c) Genome editing profile of line 24 and line 552 in T0 and three T1 plants, and compared to the reference sequences, TTTCCTTTTTGTTCATTTCCTCATTGAGGTTGGAGCGCAT and ACCTCCTCCACAGCTACCATCATCGATATATGGCGATTCT, respectively. The position of the Protospacer Adjacent Motif (PAM) is marked by a red line, the insertion of nucleotides is marked by a red border,

and the nucleotide deletions are replaced by short lines.

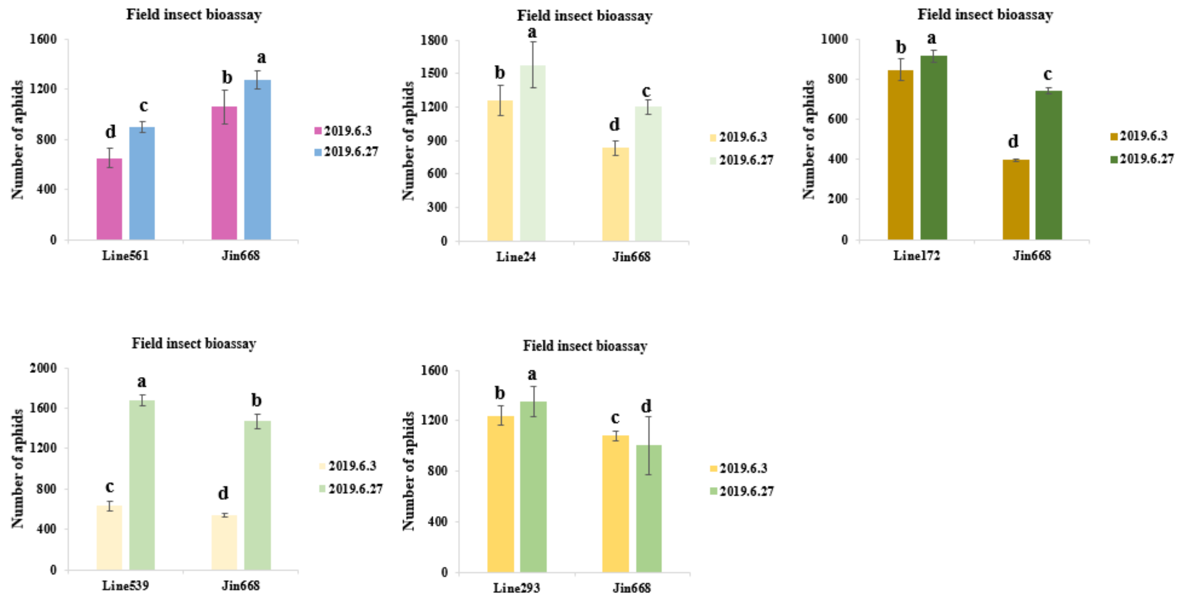

Figure S6 6

Figure S6. Determination of aphid population density of plants screened with 5 genome-edited lines with distinct phenotypes.

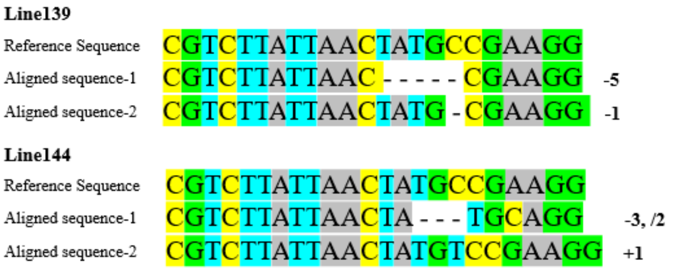

Figure S7 7

Figure S7. The editing profiles of line no 139 and line no 144 and the expression level of *GhMLP423* gene in the generated mutants.

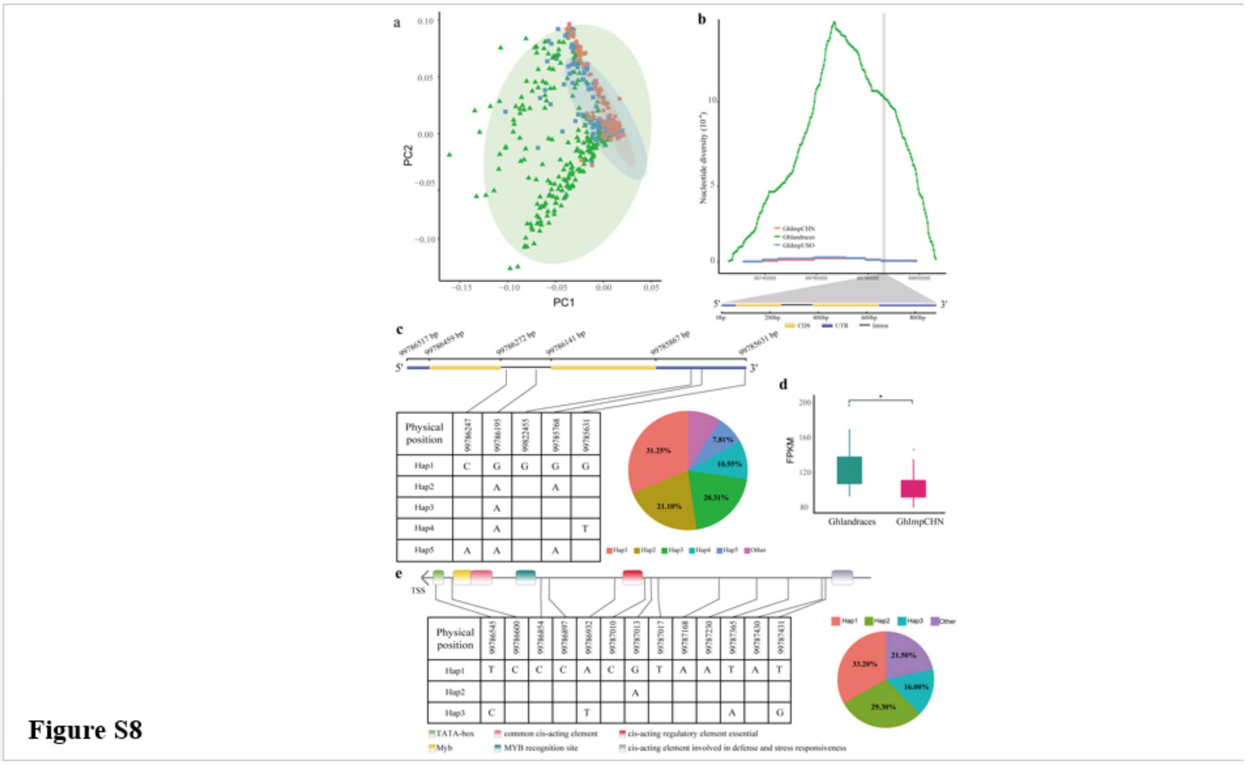

Figure S8. Evolution and selection of the GhMLP423 locus.

(a) Principal component analysis (PCA) of Ghlandraces, GhImpUSO and GhImpCHN populations. PC1 on X-axis and PC2 on Y-axis. (b) Nucleotide diversity (π) of Ghlandraces, GhImpUSO and GhImpCHN populations, with an X-axis of 20 kb upstream and downstream of the gene GhMLP423 and Y-axis represents 10-4 of nucleotide diversity. (c) The gene GhMLP423 structure and single-nucleotide polymorphisms (SNPs) variants contained in the Ghlandraces population showed the highest proportion of five haplotypes, and the blank boxes were the same nucleotides as the reference genome (Hap1). The pie charts show the proportion of the five haplotypes. (d) The expression of the gene GhMLP423, containing transcriptome data from 10 Ghlandraces samples and 40 GhImpCHN samples. * represents significant difference <= 0.05. FPKM, Fragments Per Kilobase of transcript per Million mapped reads. (e) Three haplotypes of SNP variants in the promoter of GhMLP423 gene (>5%), the blank box is the same as the reference genome (Hap1). The pie chart shows the proportions of the three haplotypes and the other haplotypes.

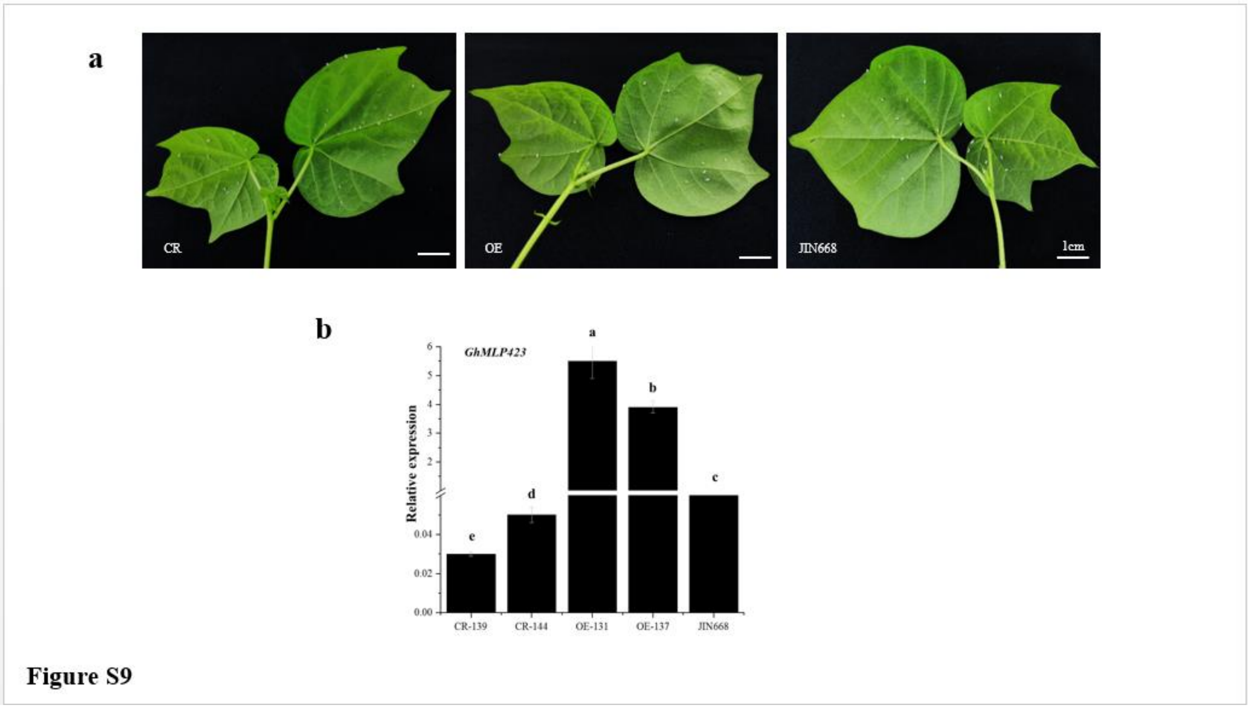

Figure S9. Whitefly bioassay and qRT-PCR analysis of *GhMLP423* gene in *GhMLP423* plants.

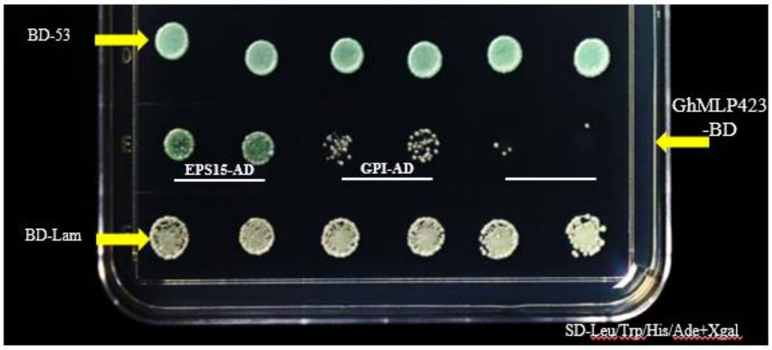

Figure S10

Figure S10. Initial identification of the possible interacted genes with *GhMLP423* by yeast library, *GhMLP423* was used as a binding domain (BD). Those genes were able to grow on the SD-TLHA+X-α-gal (T: Trp; L: Leu; H:His; A:Ade) medium.

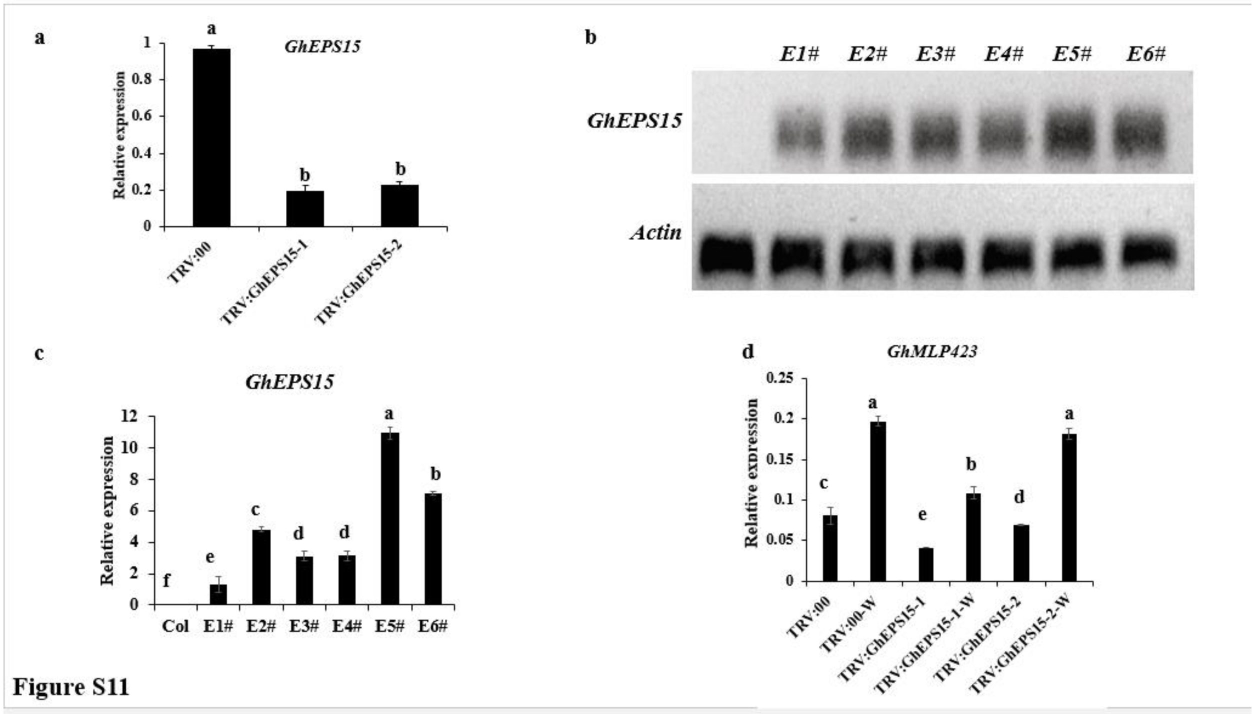

Figure S11. Molecular analyses of *GhEPS15* generated plants.

(a) qRT-PCR analysis of *GhEPS15* gene in *TRV:00* and *TRV:GhEPS15* seedlings. (b) Semi real-time quantitative qRT-PCR analysis of transgenic *Arabidopsis* lines overexpressing *GhEPS15*. (c) qRT-PCR analysis of *GhEPS15* gene in transgenic *Arabidopsis* lines overexpressing *GhEPS15*. (d) qRT-PCR analysis of *GhMLP423* gene in *TRV*:00 and *TRV:GhEPS15* seedlings.

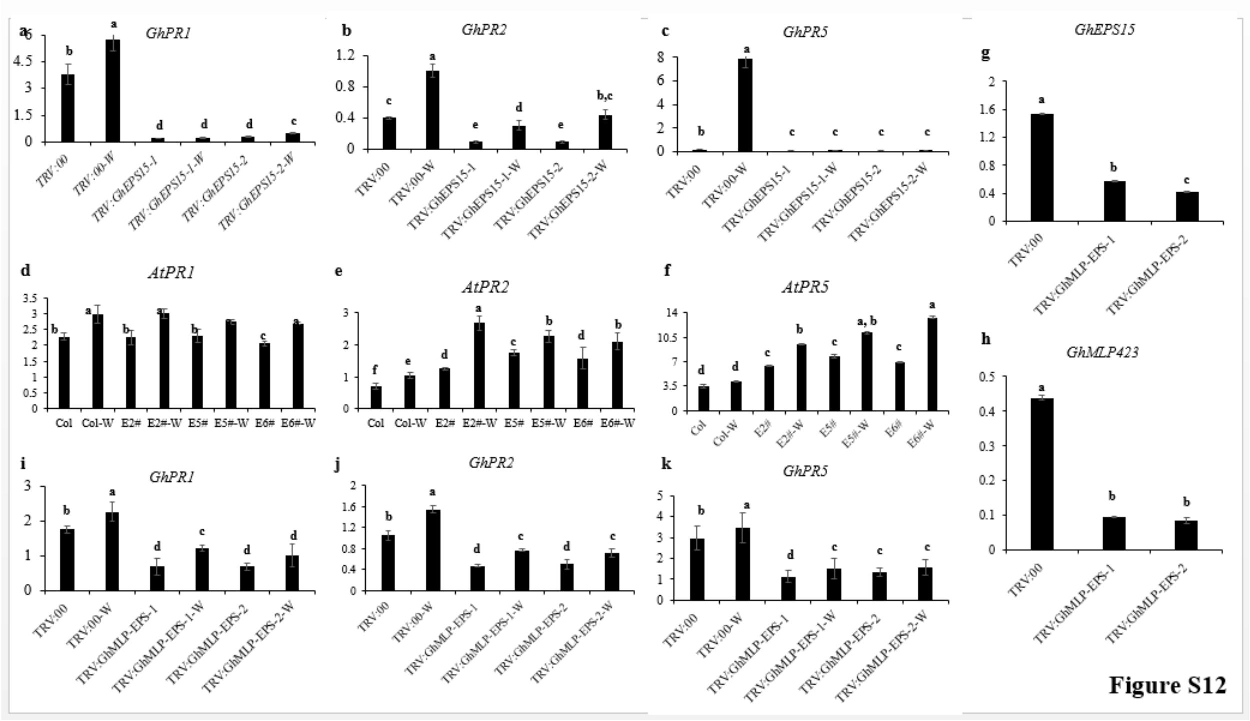

Figure S12. Expression level analysis of *PR* genes or *GhEPS15* or *GhMLP423* in different plants materials.

Table S1. List of 502 target gene IDs used for the target editing by CRISPR/Cas9.

**The gene IDs of 502 target genes**					
Gh_A01G0013	Gh_A05G3041	Gh_A10G0504	Gh_A13G1254	Gh_D05G1913	Gh_D09G1013
Gh_A01G0096	Gh_A05G3064	Gh_A10G0762	Gh_A13G1467	Gh_D05G1939	Gh_D09G1081
Gh_A01G0555	Gh_A05G3304	Gh_A10G0792	Gh_A13G1480	Gh_D05G1993	Gh_D09G1085
Gh_A01G0775	Gh_A05G3362	Gh_A10G1002	Gh_A13G1490	Gh_D05G2130	Gh_D09G1399
Gh_A01G0845	Gh_A05G3452	Gh_A10G1024	Gh_A13G1559	Gh_D05G2284	Gh_D09G1449
Gh_A01G0984	Gh_A05G3499	Gh_A10G1104	Gh_A13G1655	Gh_D05G2316	Gh_D09G1737
Gh_A01G1212	Gh_A05G3675	Gh_A10G1185	Gh_A13G1728	Gh_D05G2357	Gh_D09G1773
Gh_A01G1505	Gh_A05G3743	Gh_A10G1488	Gh_A13G1960	Gh_D05G2446	Gh_D09G2105
Gh_A01G1515	Gh_A06G0136	Gh_A10G1595	Gh_A13G1983	Gh_D05G2484	Gh_D09G2106
Gh_A01G1605	Gh_A06G0388	Gh_A10G1766	Gh_A13G2088	Gh_D05G2502	Gh_D09G2217
Gh_A01G1793	Gh_A06G0474	Gh_A10G1800	Gh_A13G2219	Gh_D05G2642	Gh_D09G2332
Gh_A01G1923	Gh_A06G0606	Gh_A10G1846	Gh_A13G2343	Gh_D05G2715	Gh_D10G0049
Gh_A01G2025	Gh_A06G0879	Gh_A10G1894	Gh_D01G0031	Gh_D05G2975	Gh_D10G0172
Gh_A02G0089	Gh_A06G0917	Gh_A10G1971	Gh_D01G0045	Gh_D05G2983	Gh_D10G0187
Gh_A02G0242	Gh_A06G1004	Gh_A10G1976	Gh_D01G0196	Gh_D05G3061	Gh_D10G0273
Gh_A02G0512	Gh_A06G1061	Gh_A10G1991	Gh_D01G0210	Gh_D05G3120	Gh_D10G0895
Gh_A02G0769	Gh_A06G1466	Gh_A10G2071	Gh_D01G0450	Gh_D05G3199	Gh_D10G0936

Gh_A02G0919	Gh_A06G1471	Gh_A10G2134	Gh_D01G0531	Gh_D05G3433	Gh_D10G1429
Gh_A02G0920	Gh_A06G1477	Gh_A10G2292	Gh_D01G0627	Gh_D05G3603	Gh_D10G1456
Gh_A02G1005	Gh_A06G1512	Gh_A11G0042	Gh_D01G0714	Gh_D05G3608	Gh_D10G1559
Gh_A02G1344	Gh_A06G1549	Gh_A11G0052	Gh_D01G0774	Gh_D05G3616	Gh_D10G1797
Gh_A02G1410	Gh_A06G1585	Gh_A11G0068	Gh_D01G0799	Gh_D05G3848	Gh_D10G2150
Gh_A02G1457	Gh_A06G1623	Gh_A11G0142	Gh_D01G0854	Gh_D06G0027	Gh_D10G2162
Gh_A02G1510	Gh_A06G1842	Gh_A11G0318	Gh_D01G0898	Gh_D06G0035	Gh_D10G2261
Gh_A02G1672	Gh_A06G1896	Gh_A11G0358	Gh_D01G1482	Gh_D06G0066	Gh_D11G0040
Gh_A02G1729	Gh_A06G1983	Gh_A11G0364	Gh_D01G1502	Gh_D06G0115	Gh_D11G0156
Gh_A03G0015	Gh_A06G2076	Gh_A11G0551	Gh_D01G1616	Gh_D06G0276	Gh_D11G0200
Gh_A03G0121	Gh_A07G0209	Gh_A11G0556	Gh_D01G1758	Gh_D06G0402	Gh_D11G0213
Gh_A03G0210	Gh_A07G0263	Gh_A11G0594	Gh_D01G1963	Gh_D06G0687	Gh_D11G0373
Gh_A03G0291	Gh_A07G0599	Gh_A11G0721	Gh_D01G2033	Gh_D06G0845	Gh_D11G0392
Gh_A03G0292	Gh_A07G0909	Gh_A11G0868	Gh_D02G0113	Gh_D06G1078	Gh_D11G0464
Gh_A03G0298	Gh_A07G0914	Gh_A11G1077	Gh_D02G0768	Gh_D06G1292	Gh_D11G0681
Gh_A03G0498	Gh_A07G1274	Gh_A11G1370	Gh_D02G0901	Gh_D06G1728	Gh_D11G0807
Gh_A03G0499	Gh_A07G1280	Gh_A11G1431	Gh_D02G1456	Gh_D06G1775	Gh_D11G0974
Gh_A03G0530	Gh_A07G1385	Gh_A11G1509	Gh_D02G1508	Gh_D06G1781	Gh_D11G0985
Gh_A03G0693	Gh_A07G1493	Gh_A11G1580	Gh_D02G1509	Gh_D06G1804	Gh_D11G1011
Gh_A03G0983	Gh_A07G1922	Gh_A11G1609	Gh_D02G1629	Gh_D06G1919	Gh_D11G1074
Gh_A03G1194	Gh_A07G2014	Gh_A11G1616	Gh_D02G1715	Gh_D06G1939	Gh_D11G1168
Gh_A03G1218	Gh_A07G2125	Gh_A11G1688	Gh_D02G1776	Gh_D06G1966	Gh_D11G1228
Gh_A03G1219	Gh_A07G2354	Gh_A11G1738	Gh_D02G1817	Gh_D06G2020	Gh_D11G1303
Gh_A03G1240	Gh_A08G0195	Gh_A11G1910	Gh_D02G1958	Gh_D06G2277	Gh_D11G1737
Gh_A03G1365	Gh_A08G0216	Gh_A11G2037	Gh_D02G2055	Gh_D07G0263	Gh_D11G2098
Gh_A03G1418	Gh_A08G0390	Gh_A11G2286	Gh_D02G2322	Gh_D07G0317	Gh_D11G2183
Gh_A03G1432	Gh_A08G0491	Gh_A11G2295	Gh_D03G0004	Gh_D07G0318	Gh_D11G2347
Gh_A03G1451	Gh_A08G0656	Gh_A11G2345	Gh_D03G0109	Gh_D07G0319	Gh_D11G2552
Gh_A03G1815	Gh_A08G0662	Gh_A11G2676	Gh_D03G0185	Gh_D07G0802	Gh_D11G2595
Gh_A03G2119	Gh_A08G0775	Gh_A11G2936	Gh_D03G0213	Gh_D07G0910	Gh_D11G3169
Gh_A03G2127	Gh_A08G0924	Gh_A11G2950	Gh_D03G0441	Gh_D07G1364	Gh_D12G0057
Gh_A03G2169	Gh_A08G1014	Gh_A11G3268	Gh_D03G0635	Gh_D07G1392	Gh_D12G0274
Gh_A04G0108	Gh_A08G1482	Gh_A12G0043	Gh_D03G0721	Gh_D07G1491	Gh_D12G0289
Gh_A04G0281	Gh_A08G1502	Gh_A12G0077	Gh_D03G0999	Gh_D07G1701	Gh_D12G0475
Gh_A04G0305	Gh_A08G1503	Gh_A12G0118	Gh_D03G1188	Gh_D07G2218	Gh_D12G0534
Gh_A04G0321	Gh_A08G1605	Gh_A12G0152	Gh_D03G1250	Gh_D08G0477	Gh_D12G1453
Gh_A04G0400	Gh_A08G1751	Gh_A12G0193	Gh_D03G1277	Gh_D08G0650	Gh_D12G1633
Gh_A04G0460	Gh_A08G1863	Gh_A12G0274	Gh_D03G1551	Gh_D08G0763	Gh_D12G1731
Gh_A04G0493	Gh_A08G1907	Gh_A12G0391	Gh_D03G1627	Gh_D08G1240	Gh_D12G1841
Gh_A04G0544	Gh_A08G1908	Gh_A12G0518	Gh_D03G1764	Gh_D08G1269	Gh_D12G2002

Gh_A04G0555	Gh_A08G2151	Gh_A12G0527	Gh_D03G1822	Gh_D08G1403	Gh_D12G2124
Gh_A04G0592	Gh_A08G2190	Gh_A12G0562	Gh_D04G0030	Gh_D08G1534	Gh_D12G2133
Gh_A04G0697	Gh_A08G2199	Gh_A12G0630	Gh_D04G0112	Gh_D08G1639	Gh_D12G2135
Gh_A04G0970	Gh_A08G2408	Gh_A12G1098	Gh_D04G0298	Gh_D08G1682	Gh_D12G2194
Gh_A04G1176	Gh_A08G2417	Gh_A12G1620	Gh_D04G0446	Gh_D08G1781	Gh_D12G2216
Gh_A05G0519	Gh_A09G0258	Gh_A12G1862	Gh_D04G0487	Gh_D08G1866	Gh_D12G2412
Gh_A05G0522	Gh_A09G0699	Gh_A12G1863	Gh_D04G0602	Gh_D08G1916	Gh_D12G2436
Gh_A05G0840	Gh_A09G0993	Gh_A12G1931	Gh_D04G0927	Gh_D08G2141	Gh_D12G2574
Gh_A05G0856	Gh_A09G1002	Gh_A12G1953	Gh_D04G0931	Gh_D08G2224	Gh_D12G2819
Gh_A05G1019	Gh_A09G1530	Gh_A12G2009	Gh_D04G1010	Gh_D08G2226	Gh_D13G0232
Gh_A05G1023	Gh_A09G1709	Gh_A12G2112	Gh_D04G1318	Gh_D08G2420	Gh_D13G0315
Gh_A05G1337	Gh_A09G1788	Gh_A12G2121	Gh_D04G1332	Gh_D08G2456	Gh_D13G0630
Gh_A05G1382	Gh_A09G1796	Gh_A12G2263	Gh_D04G1513	Gh_D08G2524	Gh_D13G0710
Gh_A05G1548	Gh_A09G1908	Gh_A12G2289	Gh_D04G1594	Gh_D08G2555	Gh_D13G0941
Gh_A05G1628	Gh_A09G1909	Gh_A12G2300	Gh_D04G1715	Gh_D08G2707	Gh_D13G1079
Gh_A05G1647	Gh_A09G2020	Gh_A12G2448	Gh_D04G1786	Gh_D09G0034	Gh_D13G1363
Gh_A05G1705	Gh_A09G2275	Gh_A12G2477	Gh_D04G1788	Gh_D09G0057	Gh_D13G1580
Gh_A05G1815	Gh_A09G2279	Gh_A12G2496	Gh_D04G1949	Gh_D09G0071	Gh_D13G1617
Gh_A05G1896	Gh_A09G2473	Gh_A13G0149	Gh_D05G0504	Gh_D09G0237	Gh_D13G1635
Gh_A05G2005	Gh_A10G0019	Gh_A13G0294	Gh_D05G0600	Gh_D09G0266	Gh_D13G1759
Gh_A05G2113	Gh_A10G0033	Gh_A13G0392	Gh_D05G1137	Gh_D09G0333	Gh_D13G1924
Gh_A05G2114	Gh_A10G0045	Gh_A13G0735	Gh_D05G1141	Gh_D09G0399	Gh_D13G1979
Gh_A05G2227	Gh_A10G0141	Gh_A13G0817	Gh_D05G1283	Gh_D09G0467	Gh_D13G2023
Gh_A05G2327	Gh_A10G0159	Gh_A13G1098	Gh_D05G1432	Gh_D09G0708	Gh_D13G2027
Gh_A05G2443	Gh_A10G0207	Gh_A13G1212	Gh_D05G1506	Gh_D09G0712	Gh_D13G2447
Gh_A05G2675	Gh_A10G0272	Gh_A13G1224	Gh_D05G1723	Gh_D09G0863	Gh_D13G2550
Gh_A05G2759	Gh_A10G0412	Gh_A13G1231	Gh_D05G1792	Gh_D09G0920	

Table S2. Sequencing results of 100 clones randomly selected to test gene coverage in a small pooled vectors library.

sgRNA target sites	Count	Gene ID	Gene coverage
GAGACTACCACACTCGTCTC	3	Gh_D05G1939	Yes
AATCTACGGTAGAGGCTGCT	6		
TACTCTTCCCTGGCCCCGTA	0	Gh_D05G1993	Yes
GACAACGTAGTAGAACAGCG	2		
TGTGGCAAGTCCGGCACTGT	5	Gh_D06G1781	Yes

TTGATGATCGCAGCCATTTT	4		
GGAAACAATCCTACTCGTTC	1	Gh_D06G1775	Yes
CCTCTAGCTGCAATACTGGC	1		
ACCGGAAATATCATCACCAT	2	Gh_D05G3616	Yes
GGTCTTTGTGGGTTCATATC	5		
GTACAGTTACTCTGTTATGG	1	Gh_D05G3603	Yes
TTTCCTGAGGGTGCAGAGGT	4		
AAATTGCGAAGTTCAACCGT	0	Gh_D05G3608	Yes
CGTCAAATGGCTGCAAGTGT	1		
TCTCTTTATATATCACTACG	1	Gh_D05G2648	Yes
ATATCTTTCTGAGATGCAAG	3		
AAGCCCAGAAAGGGTTTTCG	3	Gh_A11G0723	Yes
TTAGATCTATATTTGGGGAG	2		
AACCATTTTCCACGAAGCCC	3	Gh_A09G0258	Yes
CGTAAACCGAAGCAACTCTT	3		
TGGTTTACCTCGATGCTAAT	1	Gh_D12G2260	Yes
GGTTTACCTCGATGCTAATA	1		
CAGGAATATGATGCAGTGAT	0	Gh_A10G1595	Yes
GTTTCTCCAGTGCGTGCACA	1		
TTACCATCTTGAGGTTGCTC	0	Gh_A05G3064	Yes
AACCTCAAGATGGTAAAGTT	3		
CAGGTGGTGAAATGCTAACT	5	Gh_A06G0163	Yes
GGTGAAATGCTAACTCGGCT	2		
GGCTACACATGACTCGCAAA	3	Gh_A01G0531	Yes
GTCATCGGAGGGAGAGTATG	3		
AGTGAGCTGGTAATCGGCAC	0	Gh_D02G0304	Yes
CGACGTACCGATCGCCAACA	4		
CTTCCTTGAATTCAACAACG	3	Gh_A06G1802	Yes
GTGAATTGCACCGGAGTTTC	2		
AACCATCTCATGAGCTCCGG	7	Gh_D12G2746	Yes
ACACCATCAGGTCTATCAAT	5		
CGTCTTATTAACTATGCCGA	2	Gh_A03G1240	Yes

GTTCCCCACACTTTTTCTGC	5		
ATTTCATCGAGCTCTTGCAA	0	Gh_D04G1399	Yes
TTGGCCCAAGGCTGCTCCTC	3		

Table S3. List of all sgRNA target site sequences used for the construction of pooled sgRNAs library.

**The sequence of 968 sgRNA target sites**			
TGGACTATAGCTGGGAGATGTGG	AGAAAGGATGGCTCGCCGTTCGG	TCAGAGCTAGTTGCACCACCAGG	GTCTTGGTGGCTACTTACGAAGG
GGACTATAGCTGGGAGATGTGGG	ACCTTAAAGGCCCCGTCATGTGG	CAGAGCTAGTTGCACCACCAGGG	CTGTTTCTTGTGCTTCGACCAGG
CTATATGCAATTGCGCATCTTGG	ACGGCCAGCAACAACACCATCGG	TACTGCAATCGGGTGCGACACGG	GTGAAACTGATCACATGTACAGG
AAACTCTCACACTTGCATGTGGG	TCCGTTGTTGTTGTTGCCGATGG	GAGGAGCTACAACATAAGTCTGG	GAAGGTGGGAACATGGAAGGTGG
CTACACTATTCTTCAGAGCAAGG	CCCATACTCTTCCTCTGCTTCGG	GTGGCGTTGTTAAGACAGATTGG	ACCCAATGATGATCCTCCCGAGG
CTGTGCACGGATATTAGGGATGG	ACCTGCTCCAGCACTTCATCTGG	CTTAACAACGCCACCTCGAGTGG	GACCACTGGGTTGATGTAACTGG
AATTGAGCATCGGAGTCCAATGG	TCTCGCCACGTGGCAGTAATTGG	TACAACCCTCTCTTTGGCGATGG	TCTGTATGTGACCTCAAAGAGGG
CATCTCAACTGGTATAGTGTTGG	TGCACATGAAGATGGTGCGGTGG	GTGTTATGACGGCGTAGTCACGG	GTCCTCTCAGAGTTTCGAGCAGG
GCTCAGTTTGCTCTGGAGTCTGG	CGAAATCGTGGGAGGAACTAAGG	TCCACTCTTACTGTCTTCTTTGG	CTTTCGTTGCTGAATTGTAGTGG
AACTTGTGGTACAGTTGGACGGG	CTTAGTTCCTCCCACGATTTCGG	TCCAAAGAAGACAGTAAGAGTGG	ACATGGTTGAGACGAAACCCAGG
GATGTCTCCTGTTGAGTCTGTGG	GTATCATGTGCCTGCAACCCTGG	GGTCTGAGTATTGATGAGGACGG	CGAATCTTCGAAAAGGCCTAAGG
TCTCCGCCCCAAAAATTGTATGG	TAAAAAACTCGTCTCGGCCTTGG	GAGCCCTCTTCGGTACAAACAGG	AGCCTCTCCTACTGAATCAATGG
ATGAGAAGATTGTGTGGCCGTGG	GAACATTAACGGGACCGATCCGG	TCGATCATCGATAGCGGTGTTGG	GTTTGCTCAAACAAGCACAGTGG
AAGATTGTGTGGCCGTGGACTGG	TGAGATCATTGGGAAGAGCCTGG	TCTAGGCGATGATCTAGCAGCGG	ATTGGTACCCACACTTGCAGAGG
GATTGTTCGTGCCAATGACCGGG	CGACAAGAAACTGAAGAACCGGG	CGAGTCGGATTCCGAGATTTCGG	GATAACTGCCTTCCATGTTTGGG
TCCTGAAGGAGAGTCTTGTGTGG	GAACATTAACCTGACCGACCCGG	TGTTCTGCTGCAGCATTTCACGG	ATGAAGGCAGTGGAGATTTCTGG
GGTTGGGTCTCCTCTGATTGCGG	TCACTAATAGCAGCCACCACAGG	CAACGCCTTTGACAAAAGACAGG	TGTTGTTCAAGTTTAGCCGTAGG
CAATATGGAGAAACGCTTCGTGG	CACTAATAGCAGCCACCACAGGG	GACAAAGGAGGCATCATTTCAGG	TAAGCGAGACACCAATAAAGGGG
CTTCAGACCGTCAGCATCCTCGG	AGAGACACGCACGTTAACAGCGG	AAATGTCGAGGATGTTGAAGAGG	CTATTGCTAAGTTGTTGCCGGGG
TTCAGACCGTCAGCATCCTCGGG	AGCGGTTCCATCTCCATCAAAGG	GCAAATCAGCCAACTTAGCCCGG	TTGCTAAGTTGTTGCCGGGGAGG
GAAGTACACAGTCATGGTTTCGG	AGGCTAGGAGAGTAGTAGCATGG	TCGATCATTGATAGCGGTATTGG	ATCGGATTCTGAGTGGCTTGTGG
TGTAATTAGGCACATGGGAGAGG	TCCGGAGCATTGATATACGCCGG	TCAGGTCGATGAAGTTGTTCTGG	GGACGGCTCCGATTTGATCTTGG
TATATTCCCGGAGCCATACGAGG	GCAGTCAGTATGGATGGTGTGGG	ACGGTGTCGTTCGGTTACCTTGG	TATGACAACAGGGTGGCTACTGG
TCGTATGGCTCCGGGAATATAGG	CTGACTGCAACATAAGTTGTTGG	TGCAGTGTTTATGTTGTCTCCGG	TGACAACAGGGTGGCTACTGGGG
TCAAGTTGTCTCTCCAAAAGTGG	GCTTGAGGGTAAGAAACACAAGG	CGATGCTAGCCGTTCTGCTTGGG	GAGCATGGGATGAACTTTCTTGG
AAGCCAGTTCAGTCAATGTCTGG	AACAATGGCGGTACTTTCATTGG	CGGTGATGGTTATATATCGGCGG	GGTTTCTCCTGCTGATACACTGG
CCAATCAGCATCACGAATTGAGG	TGTGAGCGTTGAGGTAGTCTTGG	ACGACCTGATGGTTGAATCTAGG	CACCACTACTACTACCACCGTGG
TGATGAGAAAATAACCTCCGTGG	AACTCTAACAGGTGCGCAGCAGG	ATCGGTGAGTTGTTAACGTCGGG	CACGGTGGTAGTAGTAGTGGTGG
AATGAACCACCAACCAGGTCAGG	TTGGCGAAATTCAACCCGCCTGG	AAACCTGGTGAACATGACGGTGG	GACGAATGGTATGGGCACTCTGG
ACCAACCAGGTCAGGTTTACAGG	GATTGGCGTCGTAGTTGTAGTGG	GGCTTCTTCTTACGATACATCGG	ACGAATGGTATGGGCACTCTGGG

CCTTGTACTTTCACCCTCATAGG	TGATAACAGGTCAGTGCATAGGG	CATGAAAGGGGATTCACCTTCGG	ACCATTGAGGAATCGAGCAATGG
CCTATGAGGGTGAAAGTACAAGG	GTCGGTATATCCTCCAATGTCGG	GATTTACAGCCAAGGCCTCCTGG	ACCGAATCGAGTATGGGCTCGGG
AATCCAAAGGTGGGATTGAGGGG	GTATAAAGCATGCTGTGATGAGG	ACAGAATTATCTTCGCATCCAGG	GGTACTGATCTCCAATTCCCTGG
ATTTCGCAATGGATGTATCGAGG	GCACTACATTGAGCACGGAAGGG	GAGATGTCAACATGGTGGTAAGG	TTGGTCTTATTTGTGCCCACTGG
GGATACTCTGCAGCGACTCACGG	GTGGTGGAGAAGTGTTCCAGAGG	TGTGTTATCTGTAGTGATGGAGG	TGGCTCGGCTGCTGTAACTTCGG
GAACAAGAGAAACTTTCCCTTGG	AAGCTGAAAGAAGAGACCCGTGG	AACACGCGCTAGCCAGAGTAAGG	GCGGAAAAAGCTGCCGACTAAGG
AGAGGTGGTGATGGAGGCTACGG	GAAAGAAGAGACCCGTGGCATGG	CGTGAGACGATGAAAGGTCGTGG	GAAGCAAAAGGCCAACAATGTGG
CCAATCCTCTAGTTGGAGTCAGG	TTGCGGAGGGATTGATACCGCGG	CAGCTCCGGTCTGCCAAAAATGG	TGGTTAAGGTTGAAGTGGCGGGG
ACCGCTAGTAGTTGACGATCTGG	TTTAACCACTCGTTCCTCCGCGG	CGAGAAGCTATTGCAACTCGCGG	ACACATTCAAGGTGATCTTGAGG
GCTAGTAGTTGACGATCTGGAGG	CAGATGAGCTCCAAAATTTGGGG	AGGCAAAGTGGAGCATGGGTGGG	TCTGGTCCTTTGAATGCTGCTGG
ACGGAAGGGAGAGCTCAAAAAGG	CCATGCAGGACGTTGTGAAATGG	CTTCGACGACGATACATCGTCGG	AATGTGAAACTTGAAGCCTGTGG
TGTATCTTCAGAACCTCTACTGG	TCCTTCTGCCAAACATATGCAGG	GACGATACATCGTCGGAGAAAGG	AACATCGCCGGCTAATAATCCGG
TACAGTCCTTAGTGGCACCCGGG	GCTGATCTTGCTGTGTTTCAGGG	CCAGACTCACCTGTTGAGTCAGG	GTTAGTAGGGGAGGAAGCTCCGG
TAGCGTGCGTAACACTCCGAAGG	ACCAATCGAAGAAAGTTGCGCGG	CAGAAGGCTCTATTGATGGGGGG	GGGCTCAGTTAAATAGCCCTTGG
CCCTTGGTCCACTTACTTTCCGG	ATTACAGCTTCATTGCAACCAGG	CATCCCAGACTCTGAATCTTGGG	CTGAGCCCAAAACTGATCTTGGG
TTGGCTAGCTGTTCACGTATTGG	ACTAATCGAAGAAAGTTGGGTGG	ATTCCCAAGATTCAGAGTCTGGG	TAACAAACCAGACATGTCCAAGG
TCTGGCATGTGCTCGTTTGAAGG	ATTACGGCTTCATTGCAATCAGG	TTATGGATTTGGGGAGATGGAGG	TAATGGGAACCACACCACCAAGG
ATTGCATTCGGGGAGACCTTAGG	GTGTATGGATCCGCCTTGGTTGG	CTCAGATGGGGCATTTGCTAAGG	TAGGATAATGGCTATGGACCAGG
TAGTTTGCCTGTTGTGAACAAGG	TGTATGGATCCGCCTTGGTTGGG	CGGGCTAAAAGCTGAAGAAGGGG	TACCAAACGTGATCGCCAGAGGG
TTTGTAACTGCTAGTGGGGAAGG	TGTACGGATCCTCCGTGGTTGGG	GTCCCTATGATTGGATTCAGTGG	GAGAAAGAACAGGACTTCTGGGG
ATCATATCCTCTCTCGATGGAGG	ACAGATCCACAGGAATTCGAAGG	AGGGACATAATCCTCACACTCGG	TGGAGTTGGGGTTCTGTGAAAGG
CATTACTCCTCCATCGAGAGAGG	ACGACAGCAACAGCAACGAAAGG	CAGCTCCAGTCAGCCAAAAATGG	GAGTTTCGTATTAGTTGACCTGG
ATTGTATCGAAGCCATCTGAGGG	CTGTTGCTGTCGTGATTCTTTGG	CCTGATGTTGGTTCCATTTGAGG	TGAATAATGGCTCTCGCTGTTGG
TTATGTGTGTCGCATATGTGGGG	CATCCCTGATTCTGAATCTTGGG	CTTGTTGCTGTTGCTGTTGATGG	CCCTACTGTAAAAGTACCATTGG
AGGTTGATACAATCCCTGCTTGG	GGTGTGATTATGGGGATTGTCGG	ATAATGACTAGGATGAGACCAGG	ACAGTAGGGGGACGCAAAACAGG
ATCCAAAGCTCATGCAGCGACGG	GCATTCGTTAAGGATAAGAGGGG	GAAATTGATGTTTGCGTAGCGGG	GTAAGGAACCCCTCGGCATATGG
AAGGCCACTCCTAAGTTTCCCGG	ACAAGCTCCTCAGCTTGTTGAGG	AGGAGTACCGACAAGCAACAAGG	TGCCGAGGGGTTCCTTACAAGGG
GTGATTCTAGTGATTGCCTCGGG	GCTGCTTCAACCAGCTGGTTTGG	GACTTCTGTTGAATCTGCCGAGG	GGCCTCTGCATCAAGAGACATGG
TGTTCCTACTGAAATGCAACCGG	CTTCAACCAGCTGGTTTGGGCGG	GAGACTATATTGGCTGCTCACGG	CCAACGACCTTATCAGCCGTTGG
ATACGCCATATGATGCTTCTAGG	ACTTGCCGGAGTTCGATCAGAGG	TATGTTATTGCCGGGGAGGACGG	CAAAACAAACGGCGCAATTATGG
GAGAGATTTGACGGATCAGATGG	ATATCTCCTCCTTAGCTCCGCGG	CTGCTTCAACCTGGTTTGGGCGG	AGAAGGGATACGGAGAATTTGGG
ATGAGAAGATCGTTTGGCCATGG	GAGGATTTACCATACCTGGATGG	CTTAAAGATATTTCAGCGGGTGG	CTTCTTCTATGACGACGGTTCGG
AGCCCTTGTCACAGAGATGCAGG	TTACCATACCTGGATGGCGACGG	TTAACCGGAGTCGAACCAACAGG	CGAAATCGTTGGATTCCTATTGG
CAAAACCAGGTCTGTACAGGTGG	CCATGGCTCCATTGACAACAAGG	TAACCGGAGTCGAACCAACAGGG	ACCTACCTCAACAGAGCAAAAGG
GTATGGAGTTCATCACTACGGGG	GGGGAGCTTGGCAAGTATTGAGG	CATGGCATCCCTGAACCAACCGG	TGCTCTGTTGAGGTAGGTGCTGG
TCATCACTACGGGGCTTGAAAGG	GAGGAGAAAAGTGGCTCCCTCGG	CGATTCTCTGGTAACGAGTTCGG	GACGATCCCATTATGGAATTCGG
CTACTATGGCTTGGCCAGTCAGG	GCCTCAAATGAAATCTCTTGCGG	ATTTGAGGCCAGACATCAAAAGG	CTTGCCGGCATCAATGATGTAGG
AGCTTGTGGTACAGTTGGTCGGG	ACCATATCCTAGAACCTCTTCGG	TCAAAAGGGGGAATTTCAGCAGG	ACTAGCCAAGGAACAGCAGATGG
AAACACTAATTCGAACCCCGCGG	TCGAAAACATCACTTCTGTGTGG	TCTATGGTGGGTACAAACAGTGG	TTTCTTCGAGCTCCCACCACAGG

ATAAAGACACAAGTGACTCGAGG	AAAGATCGTCATCGGCGCTGCGG	TTAGAAGAGGAAGCGGCGGTGGG	AGCTTCCTTACGGTTTCATGGGG
GATTACGGTATTCCGCAATCAGG	CCTCGGAAATAAATCCATGGTGG	AGCCAGAATATGCTGGCTAAAGG	CCTCCCACAAGGCATGCTTACGG
GTCTCTACGAATCCTTCAAGTGG	CCTTTCCATCAAGTTTCGACGGG	GAAGAATGAAACCTCGGCGGCGG	TTGTGAACCAAAAATCGGAGTGG
TTCAGACTGTCGTCATCCTCGGG	TTTCACAGGGTGTTCAACGGTGG	GGCTTTCCGGAATCTTCACGTGG	ATGTGGTTCGAACCTATAGTAGG
CTTCAGACTGTCGTCATCCTCGG	CTTGCTCACTGGTGGAAAAGAGG	CGAACCTACAAAACCGAAGGGGG	TGGCCATCCAATTGCCATAAAGG
CTTATGGACTGTGAGGCTGCCGG	GTCGATAATGAAGGAACTTGAGG	CATCAAGTTTCGCCGGTTACTGG	AAATTCCCTGTTCCTTTGCTTGG
GAGGCTGCCGGTATCTTAGAAGG	ACTACTTCACCAGATGATGATGG	GCGTGAAAAAAGCCAGTAACCGG	GAGAAGCTTCAACATGTACCGGG
AACCTGTGGAACGCCACCAAAGG	ACTTGGTCTTGGTGAGAGGTTGG	TAAGCCACCTCTCAAGGTGGCGG	TAAGGCTTAACCTCGATGCTTGG
AACGCCACCAAAGGCAAACGAGG	AAGCCTCATACAAGTTGAGCAGG	GGAAGTAAGATAAGCCTGGCAGG	TCTGCGGGGAAGTGATAAAGAGG
ATTGGAAATGATAACCAGCCAGG	GTACCCGCCTAAAAGTCCAGCGG	CAATCTTCTGCAGTCCCTATAGG	CTCCAATTGTTTGTTCCGTTCGG
ATTTGTCCACACAGGATTGCTGG	AGAGCAGGTAGTGGGTACTGCGG	TCTTCTGCAGTCCCTATAGGAGG	AAAACATCGAGTCCGGAATCCGG
ATGAGCTCAACATGCTTCCTAGG	AGAGGATCTTCCCACAACTTAGG	ACACTCTGAAATTTCCGACACGG	TGCCCGCGATGACGTTTCTTGGG
CAACTGGCTGCCTTGTAAGTGGG	CGAAAACCCTGCAATCTTTAGGG	CTCTGAAATTTCCGACACGGTGG	GCATGGAGACCGAATACTTGCGG
AGCTTTAAAGCTGACCTGGCTGG	AAAACGAAGGGAACTACTCTCGG	AGAGCTAGAGCAATTCACCGAGG	TCCATGCTCGAGTAGCTTCCTGG
TTTAAAGCTGACCTGGCTGGTGG	TTCTCCCGCTTTCATATCGCCGG	AATTCACCGAGGCTGTTGACAGG	GTTTCGGACTAACATGCCACCGG
TCACGAAGAGCTCCCTTGAACGG	CGTTTGGAAGGGCTTCCCAATGG	TCTCAACAACAGCGCATCCAAGG	AGGTTCATGGGTGACGATGGAGG
AAGTAAAGTTCACCAGGAGCAGG	CACTAAAGTTCAGCCAAGCAGGG	CTCAACAACAGCGCATCCAAGGG	TTCTATAGATAGTGGCTTCCCGG
CCTGAAGAATGTTGTATCGGTGG	GTGGACTGTGAGGATGTTTCAGG	GCTTCGTTACTAAGAGACCATGG	ACCTGTTCAAGAGGTGATTGTGG
TATGCATCCAAAAGGGACGTCGG	TGAGGATGTTTCAGGCTGTATGG	TGATTCCAATGCTTCCTCGAGGG	AGGCCAAGCAGTTCATAAGAAGG
CTCGAAAAGTAGGTGGACCAAGG	TCGGCGAATCATCCGACTTGCGG	CTGTTATCAGCTGGACACGACGG	GGCCAAGCAGTTCATAAGAAGGG
TCGAAAAGTAGGTGGACCAAGGG	GAAAGTCGGGGAAGAGTGATCGG	CCAAAACAGCCACGTCATGGCGG	TGAAGAACTGCGGTCTGATGTGG
CAAGGTGGAAACATGTATGTGGG	TTGAAAGTAGTGTACGGCGACGG	CTTCCTTCGTCTCACCAAACAGG	TCATTTGCAGTGGAGGCCATTGG
TTCTTGCCCTCAAAGCCGTCAGG	TTAATGGAGTATATGGACGCCGG	TTCTCCCGCGTTTATATCGCCGG	GTTTCCCCTATGGTCATGCTTGG
CCCCGAGATTTGGAACAGAAAGG	AGCTTCAATAGTTCTTCGCAAGG	AGTACTTGTTGTCGAACGTAAGG	TACACATGCTGTTGGATGGTTGG
CCTTTCTGTTCCAAATCTCGGGG	TCAATAGTTCTTCGCAAGGCAGG	GTACTTGTTGTCGAACGTAAGGG	AGGTACCTCAAGTGGCTCGTAGG
CAACACAGTCATCACCGTTCTGG	AAATCCTTCAACGGGTTTCGAGG	AATTATCCATGTTAGCACGCTGG	GGTACCTCAAGTGGCTCGTAGGG
AACACAGTCATCACCGTTCTGGG	AAATCCTCGAAACCCGTTGAAGG	TATCCATGTTAGCACGCTGGCGG	ATTGATGAAGAGAATGAGGCTGG
GCATCAACCGTAATGATACAGGG	AGAAATTCCTCGATCGCCGCCGG	TCTCTGTCAAGCTTGGTTCCCGG	GAATTAGGCACGATTTGCAAAGG
GTTCCTCCACATCTTGACCTTGG	TTCCGGATTCATCTTCGAAATGG	TGGCGGGAAAACGGTACTACGGG	AGCTTGTTGCATGGGCCAAAAGG
CAACTAGTGGTTTAGCATGTGGG	ATGAACGAGATCCGTTACCGCGG	AGAGATTCCGATGGATACAGCGG	GTCTCCCAGCTCTCTTTCCAAGG
TACTTCAAACGAGCACATGCTGG	TCCGGATCTCAGCTGCGTAACGG	GTATCCATCGGAATCTCTTGAGG	ATGAAGTGGGGGCAGAGTATGG
GCGAAAATCACCTGCAATCCTGG	GAGCAACTAACGGCAGAGCAAGG	TTGAAAGTCGTGTACGGTGACGG	GATAGAAACCCTGAAGCTGTTGG
CCTTGATGAGCCTGCTTCGTGGG	GTACATCCATTACTCTCGCAAGG	TTAATGGAATACATGGACGCCGG	ATTCGTAAGTGAGGGATTTGAGG
TAGTTCACCTGGTGTGAACAAGG	GTGGAGTCAACACCTTGTCCGGG	TGTTAGAATATGGTGGCCCTAGG	ATCCTTGGCCTCATCGCATATGG
ATACCAAACCAGGGCCGAGATGG	TGGAGTCAACACCTTGTCCGGGG	ACGGCTGATAGTATGGCCTAGGG	TTACAGGAGGGGAAGTACCGCGG
AGACTTCAAACGTGCCGAGTAGG	GTTGAAGCGAAGACGATCGGCGG	AGCTCGTTAATGTCCACCAGCGG	GCGTAAGCATCTGGATAGTCAGG
CTGAACTTCATCCATCTGTCTGG	ATCCGGAAGCATCAGCAACCCGG	ATGTTCAGTGGCAGTGCTCGTGG	CACCTCGCTTACAGCTAAACCGG
GGAAAGCTGGGAGCTATGAAGGG	GAAGTGTACCCAAGGTTTCAAGG	ACGAGCACTGCCACTGAACATGG	ACCTCGCTTACAGCTAAACCGGG
GAAAGCTGGGAGCTATGAAGGGG	TAGGAGAAACATCATCCCCTTGG	ATACTAATAAGGGAACTGGCTGG	CCCCCATCCCCATATGCAAATGG

TCAAGCGGCTGCTCACACATTGG	ATGGGTCTTTCAGATCACTACGG	ACTAATAAGGGAACTGGCTGGGG	GCTATCGTAGTTGTCTGGGGGGG
TCTTCATGTTGCAGCTGAATGGG	TGGGTCTTTCAGATCACTACGGG	CCAATCTTACCTAAAAGCCTTGG	AAATCTCCACAAGGCGGTGATGG
AGAACTGACTGACCTCCGATCGG	AGCAGGGGAGCTGAAATCCAAGG	GGTACATGTGCGTCATCGGACGG	GGCTGTGAACAACTAGAAAGAGG
GGTCTCCTTGTGTTCTGTCACGG	CATTTGCTGCCTTCACCTTAAGG	CGACTTATTGCGCTCTTCCACGG	CTGTATGGCAACTCCAGGAAAGG
GTTTAGCCCCAAATTGACTCGGG	TTATGCCACAGCCAAAAGGCCGG	ATGTGAAGGATCTGATGCTTTGG	GATGAACCCCCAAATCAGCCCGG
TTTAGCCCCAAATTGACTCGGGG	CGGAATGACGATGGACTGAATGG	TGCAGCACCCAGTTGTTCAGTGG	ACAGAAGAAGCAGCTGTGAATGG
CTCGGGTCACAGTCCTTGTTGGG	GTACCAAAGAGCTCGACCTTTGG	GGGGATCAATCCAGAAGACTTGG	CAGAAGGCATCATGCTGGTTGGG
TCGGGTCACAGTCCTTGTTGGGG	TCCACATCTTTGTTGCAATGGGG	GTGAAAGCATTAGTGCTTCAAGG	GAAAGCTTAGACGGCGATCGAGG
ACCAATATATTCTGCCCCCCCGG	AGATTGGTAGTAGGCTGAGTTGG	GGTTTGCCAGAGGCTTTCTGAGG	AACAGATCTGCTGCAAATCTCGG
AATTCCTTCCATCTCCGGGGGGG	GTAGTAGGCTGAGTTGGAATAGG	GGGATCATCATCCTTGTTAATGG	GCGATAGCAGATCGGATAATTGG
AATACTACTCCTGGTGCCTATGG	AGTGTGAAGACAGGTGTGAAAGG	CAGACATGGCTGATGAGCATGGG	TCGGATAATTGGTTCGTCTCCGG
CAATTCGGTTAACAGTTGCATGG	TGGCAGCTTTGTTCGCACTGTGG	AGATGGAAGATCCTTTGAGAAGG	TCGGAGCTCCGATACCGAAAAGG
GGCATTCCTCGTGTGTAGCTTGG	TTCGTGGTCCTTGTTAACGAAGG	ACATCCTTGCTCCCTAGTCCAGG	CCTACTAACCCGACCCGAAACGG
ATACCTTTGGAGGAGAACCGGGG	GTCCTTGTTAACGAAGGAAATGG	GTTGGTGATGGAATCTGTTGCGG	AAATCATGTGCGGAGACATGAGG
AAAGCCTAAGATCCTCTTGGTGG	GACGTCGATCCAGGTCTCAAAGG	TTCGAAACGGCGAGGTTGACAGG	TGTCGAGGACCGTGCCTAAAGGG
ACTGTTGTTTCAGGTAACTCTGG	GAAAAGGGGCTGCATTTGTGAGG	CAAGTTAACTGCCAAAGCGGCGG	GGTAAAGCTTCAACGAGCTCCGG
ATATCTGAGAGGTCGTCATCCGG	GGAAAAGACCGGAGAATGCTGGG	GAAACGGGTGGGAAAAGACCGGG	AGCTCGTTGAAGCTTTACCCTGG
TATCTGAGAGGTCGTCATCCGGG	TGTTGAAGCATGTAGGCGTTGGG	CGTCGCCTGGCCAATTTATTGGG	CCAGTGGTAAACCGAGTGTGTGG
GAATGGATCTATGGCCCTTCAGG	AATCTAGCAGCCAGATACGCGGG	ATTGTTACCGAAGCCACCGGGGG	CAGTGGTAAACCGAGTGTGTGGG
AATGGATCTATGGCCCTTCAGGG	TAGTGGATATCCCGCGTATCTGG	GGTTATCTCCATTCCCGCACTGG	CGAATTATGTTCATTCGCTCAGG
ACCGAACTGAATATGTCGCCGGG	GTCAAGGAGAAGCTATGCCTCGG	CGACCTCTTGCTGTAGACGTAGG	TGTATATGTTCTTAGTCGCGGGG
CCGAACTGAATATGTCGCCGGGG	TTCCCAATTCCAGGCTCTTGTGG	GCAAGAGGTCGTTGATCGATTGG	TTTGATTGCCGAGGAACCTCCGG
AAAAGCTTCTCGGAAGAGACCGG	TTGTTGCTTACAGTCCTCTGGGG	AAACCGGCGGCCTTACCATACGG	GTCAATCGAACACCCAAATCTGG
TCTGAGTACGTGTGGAGATTTGG	CTAGAATCCATTGCTTCAGCAGG	TTTCCGTATGGTAAGGCCGCCGG	ATGTACGGGGATACTTTGCATGG
CGAGATTCTTTGACGCTTTGCGG	GCAGCAGCATCGTGGAATTTAGG	GTTCTCAGCGAAGGACTACCAGG	AAAGGCCTAGAACATCGCGACGG
CCCGGCTGGTAATACCTTAGCGG	TGGGCTATTGACACACACTTTGG	ATGCTTCAGCAATACCTTCAAGG	TCGCGATGTTCTAGGCCTTTCGG
GAGGAGTTGTAAAGTTGGTGAGG	ATCGATGCTGTGCGGATAAAAGG	AGGTATTGCTGAAGCATCTGTGG	ATGTTCCCATGGGAGGCTTATGG
AGGAGTTGTAAAGTTGGTGAGGG	ATGTCAGTACCAGCATCTAAAGG	GTCGAGAAGGAAAGGCAATTCGG	TTCAAGGTTCACTAGCAGCACGG
AACCAGAGCCGTCCAATGACTGG	AATTCTTCGTCGGCGGAAATTGG	AGAAAGTTGCATTGCCTGGAAGG	TTGTGCTGTTGTTGAAGTCATGG
AAGTTCATGAACCGGTCTTTCGG	TGTGCAGCGACTTGGAACTCAGG	TCTCCAACTTGGCCAGCTCTGGG	TGTCATACGAAGTTGGAGCGTGG
CGACGACTCGTGCTTCACATCGG	AGGTAGGAGTCAATAGTGTCGGG	ACCAATTCAGGAATCTTCGATGG	TCCTTGCTTAGCGGAATGGTTGG
GGAGAGCAAACGATTCATGTCGG	CAGAACAAGAGAACGTGAAGAGG	GAAACGTATCCGAAAGCCAACGG	ATGGAAGCTCAACTCATCAGCGG
GGCTGAGTACTGTTCAACTGTGG	AGCCGTTCAACTTGCGCTGAAGG	TTTGACTGGCAAGTGCCAGGGGG	ACATTCCCGGTATCAATTTCAGG
CAGCCAGTTCCTCTTGTTGCCGG	TGACTGAGCTGCTTGATTATTGG	AAAGTTTAAAGGCGCTAGGTGGG	GCAGGCATGCAATGACGAGACGG
CCTTTCGAAGAATCTCCGTATGG	GCAGACTTGAGACCGGTGAAAGG	TGCAGCTTCTTTACCGAGAATGG	CAACTCGACACAAATGGGAATGG
CCTACTCACAAGCCCCTTAAAGG	TCAAACTTCTTCTTCCCAGTAGG	AAGCATTGTGTCAGCTTGTTGGG	CATTCCCATTTGTGTCGAGTTGG
GCGAATTTCCGCTAGGACTGGGG	CCTCTCTTGTCTTTCCTGATAGG	CTAAAAAGGCCGGTGGGTCTAGG	CGTACCGCAATAGTTAATGCAGG
GGATACCGAGATCTTGGTTGAGG	ATAGCAATGAACTACTCGTGGGG	TGCCGTTTCAAAAATAGCACCGG	ATTGGCTTGACTCGCAGCAACGG
AAGCTCGGACAACAGCTTCCTGG	AATGAGGTGCACCTTGATAATGG	TTTGAAACGGCAAGGTTGACAGG	AAGTGGAGTGGATTCAGTTCGGG

TGTGAATTTCCAGTAGGACTGGG	TTATCAAGGTGCACCTCATTAGG	AAGATGTAGAGATGGGTTCCTGG	GAAAAGCTCCACAATCCCACAGG
TGGAGAGGAGACGCAGAGTTTGG	AGCGGCACATTGGTCCTTGATGG	ACAGCATCATGACGGAGCTATGG	CAAAGGAGCTTTCATGGGTTTGG
CTCTTCCAATATGATGGTGATGG	ACTTCAGAGCCAAGTCCTTGAGG	GACTGTGTTGACAACTTCCCTGG	TTGATCTTGAAGGAGGCGGCGGG
ACTAGGCTTCGACGACTACATGG	TTTCCCAGCAATGATGACAGAGG	CAGGATGCGGTGCATAATCCGGG	CAACTCCAACCTGGTTTCCATGG
TGAAGCGCGGGAATGCATATGGG	TTCATCGGCTACTACAATGAAGG	ACGTGTGTTTGCAGGATACTTGG	TGCATGTTCAGCACGTAAACCGG
GTCGTTTGACCAACGTTGTTAGG	GGTTATTTGCACTCCAGAGTTGG	AGACTTGGCCGATGGTGTACCGG	ATTGCTACCATAGTTGCTCCCGG
CATGGATTGGTCTCAGAATTCGG	TATGTCGTACACAACATCGTAGG	GGCTGAATGCGAGGAAGATGGGG	GAGGTTTCCGGCAATGATCCGGG
GAGGGTAAAAGCCTAGATGATGG	CCTTCACCCAAGCACAGACATGG	GCACCGTTTCAAGCATAGGTGGG	GCTTGGCATCTTCTTTGCGAGGG
CAGGTTGAGCTCGATGATGATGG	TCGGTAACCATGCCCATTAATGG	GATCTAGAACTTGGAAAGCCAGG	AAGATGCGCCGGAGTATGTGTGG
ATCATCGTCGTCGTTTAAAGAGG	TGCTTCGTTCATTCCGACGACGG	CATTGAGTGTGGGAAAAGCGGGG	ACGGGGTTGTCGTGAAAATATGG
ATCGTCGTCGTTTAAAGAGGAGG	CTCAAAGATTGGGGAGCACATGG	CAACAACAACAGCACTGTTGTGG	ACGAATGGGTGCATGTCTACAGG
CTGCTGGTGTACGAGAACAAAGG	GCAAGTTCGGTTGGTCCATTGGG	GCAGATTCGCCTGCAGAATCTGG	TTAGAAGCTCCAGGCCTCGTTGG
GAAATACTCGGTAACGGTGGTGG	CTCGGAAAGTACACTGGCGAAGG	CAGCATTAGTCTCCCATGCCCGG	AGCATGGAGGCATCTAGCCTCGG
TGGGATGTGCGACAATAATTTGG	ACTGTCCACGAAGACAGATAAGG	CTGGCTTAAACAGCAACTCCTGG	AGATCACTCGCTTCTGCGTTGGG
TCTGATCCTGATGAGATGACTGG	AGAGAAAGCACCACCACCGTGGG	GTTGTTGTTGGCTAAGAATCCGG	CTTAATTTGGGTGGTTTACCTGG
CTCGAAATTCCCTAATGAGCTGG	TCTTCAATCCGACAATGGTCTGG	GCTCGGAAAAGAGCTCGAAGAGG	ACGGTGCAGTCGACAAGAACAGG
AGACACTGGGATCGTAGATTTGG	TGGAGATTGAACACGTTGAATGG	TGGCAGCAGTCACAAGTATTAGG	CAGTTGTTGTATTAGGCGAGTGG
CATCAGCGAGTAACTGGCTTTGG	TTAACTTTAACACACTCGCGTGG	TACTAGGCCTGTTCTTGCTTCGG	GGAAATGAATTGGGTCCGTCCGG
GTGTCACAGGTTCTTCTAGTGGG	ACAGAAGAGATGTCTTTCGCAGG	TTATAAGTGGCGGATCAACTAGG	GTCCGGTCTTCCGCCATTAATGG
TGCTACCAATTTGCAGTCACTGG	GTGGATACGGAAATGGTGGAGGG	TAAAACTTGGGCTGCGAAAAAGG	GTCTTTCTTTATGGGCGCCGTGG
TTGCAGGTAAATGCCAGGTGTGG	TACGGAAATGGTGGAGGGTATGG	CATTCTTAAGTGATGCGGAGTGG	TCTTTCTTTATGGGCGCCGTGGG
CGAGATCAAGCACCAGATCAAGG	ACACTCCATTATTGGAAGAGCGG	AGTGATGCGGAGTGGGAATAAGG	GAACTGCAACGCAAATCCACTGG
AGGCAAGAAACAAACCAAGCCGG	ATGTTGCCCGTCACTGTAGTTGG	GTCCGACAATGGTCAATCTGTGG	TGGGTAAGATTTCCCATTGTGGG
GTCGAAAGAATCCCAACTCCGGG	CAAGAAGCAGAAGGAGGGAATGG	TGCATCCATATGTTTCTCCGGGG	TCAAGGTCGGAAGGAAGAGCCGG
TACCAGTAATGGGGGTGTACTGG	AAGGAGGGAATGGCGTCGAAAGG	CTCCTTACGGACAATTCTGGCGG	TTGATGGGTGTTGATGGCGTTGG
TGATGATAGGTTCATCGTTGTGG	CAATGGATTTAAGATGCCAGTGG	TCCTTACGGACAATTCTGGCGGG	CGTAGAAAGGATGGGGCAGTTGG
CAAGCATATTAGAAGACGAGAGG	GAATTACTACCGTGTTTCGTTGG	GGTATCCACGAAACTTCGAGAGG	AGAAAGGATGGGGCAGTTGGTGG
AAGTTCATCGTTGTCGGTAATGG	TACCCCCGCCATTTCATCGTTGG	CGCATCCTCTCGAAGTTTCGTGG	TCCTCAGGATTCCCAATATCAGG
AAATGCACGATCGTGATCTCTGG	ACCCAACGATGAAATGGCGGGGG	TCAGACAACAGGAGAAGGACTGG	CACTTTGGCTCAAGGTCTTCCGG
TGACGATGATCGATATATCGTGG	ACAGTGACGGGCAACATTTCTGG	TCTACCTGGTCCAATATTGCTGG	CACAGTACCAGTGTGGTTAATGG
GATGACACCTAGGATCTTAGCGG	CAAAGACAAGAAGCAGAAGGAGG	CCAGCATCTGCTATCCCTGATGG	CAGCTACCATCATCGATATATGG
CACCTAGGATCTTAGCGGCACGG	GAGATTGAAGGAGAATTTCGAGG	GAAAGATCGAACTTTGGAGGAGG	TGGTATCCCAAGCCCTTTATCGG
AGGGAGGGTTATATGCAGTTGGG	CCGCCATTTCATCGTTGGGTCGG	ATTCATCTGCCGCTGGGTTGAGG	GGTGAGATATGCATGAAAGCAGG
TCGAACAACCATCGATGGATTGG	AAAGCATTCTCGCTGTCCTCGGG	CAGCTTCACCCCTGAAGAAACGG	GGATCGGCTTCAACATTGGGGGG
CTGTTGCTACAAGGCATAGGAGG	CCACGGAGACATCTCTGAGAGGG	ACCGGTACATTAACTTCTCTAGG	TGCATGAAGATCCCCCAAGAAGG
GGTCTGAAAAGGCCTCGTGTAGG	TTGAGGGCAAACAACTTCCAGGG	GTTGAAGGAAAGGGAAGAAACGG	GACGAGGAAACGCCGTCATATGG
CAAAGAACCCTTTCCCTAACGGG	TCGTTAAGAGCAAGGACCCCTGG	GAAGGAAAGGGAAGAAACGGAGG	GACGGGATACCATCTTCAACTGG
TTGCTGGTTCTCTCTTGCATGGG	GGGTACCATCAACATCTGTTAGG	TACTTAGAACGAACCGAAGAGGG	TGTTGCATTGAGGCACAACCAGG
TTCAACTTAGCTGCAGTGTGAGG	TGATGATGTTCGAGACCTCATGG	ACACAGGCTCCACCACATTTAGG	GAGGCACAACCAGGATCTTGCGG

GAGAACCGGCAACAGTTATGTGG	GCTGGGCTTCAATTACAGGTGGG	TCTTTCGTTTCCCTCACCATAGG	TGGGAATTAGAAGAGCTGTACGG
AGAACCGGCAACAGTTATGTGGG	CTGGGCTTCAATTACAGGTGGGG	CTCAGCATTGCCATGGTCAGCGG	TCCGAACGTTCTTTCTCCGGTGG
TCATCACAATCCAGGGTTGCAGG	AGGAAACAAATTGGCTATGCAGG	GCTTCAACTCACCATCATATTGG	TACGGCCACCGAAAAGGAGACGG
GGTTGCAGGCACATGATACCAGG	GCACTACTCCAAACTAACCAAGG	AAATTACCGGTCGGTGACGACGG	AGGGTTCGGCAGTATATTCTTGG
GGTTGCAGCCACATAATACCAGG	ATTCGGCGTCCCGATTATCGCGG	ACCACCGATGATGCTAAACCCGG	GACAGCTGTAGCTCTTTGGATGG
GTATTATGTGGCTGCAACCCTGG	AGAGACCAAGACAACGACGACGG	AAAAGCCCCACGTTAACATCGGG	TGGTTCCCTCTACAAATCAAGGG
CGTTTACTTCTCTGATGACATGG	GATCCGGTGGATATGGTGGAGGG	CACGTTAACATCGGGACCATCGG	ATAAGTATCTCTAGCGCTTCAGG
GCTGACGGCGCACACAATTATGG	ATCCGGTGGATATGGTGGAGGGG	TTCCTCCTGTTGATTGTCGCTGG	TAAGTATCTCTAGCGCTTCAGGG
GGTTAGTACATCTACCCACCAGG	TCACTACAGCTGATGAAGCGAGG	TCCTCCTGTTGATTGTCGCTGGG	GTGGCCTCAATGCAAGTATTCGG
TTACGTGTCTCCCTGGATCGAGG	CTGAGGTCTTAGCCTTCCTCAGG	TCTTGAAATCCAGATTCCACAGG	ATCCCTCTCAAAGGCCAAAGCGG
TGCTAAGTCAGAAGAAGCTGAGG	AAATGTGGTACGAGCAAGCACGG	TGTTCCAGGCTATGATGTAGCGG	ACTGGCACTATCAAGTTCAAAGG
AGCGTATTCGCTCCCAGATGAGG	CGAGTTAACGTACAACTATGGGG	AGGCTATGATGTAGCGGGAGTGG	GCACTATCAAGTTCAAAGGAGGG
GTTTCCTTGGATCGCGGAGATGG	TGACCCAGAGAAAGATGCAAAGG	GAGGCAACTCATTTCCCAAACGG	AACTTTGAGAGATGGGATACTGG
TTACGTGTTTCCTTGGATCGCGG	AACGGGCAATGTCGAATATTTGG	TTTGGGAAATGAGTTGCCTCCGG	TATAGGCAGGACGCAACCATTGG
CGGTGCAAGTCGTAATATGTTGG	GATGACAAAGCTGCTGAGGCAGG	ATGGGGAATACAGCCAAACTCGG	GAACGCCCAATAATTGTCAACGG
AGTTTAAGGGACCTGAGGTTCGG	ATGACAAAGCTGCTGAGGCAGGG	TTTGGCTGTATTCCCCATGCAGG	CGACACCGTTGACAATTATTGGG
CAGCTATGCTAACATTACTGCGG	TGTCTCTGTGGTTGACCTCACGG	CTGCAAAAGTTTCGCGGTTCAGG	TCTAACAGCTCGCTCTCTCCTGG
CCATCGGATATTGCAGGACAAGG	TTGGTATCGACTGACTTTGTTGG	CAATGCTCCTTGTAGAAAAGGGG	CTCGGAAGCCTGGATTCATTTGG
CTCCAAAACCAGCATCGAGTGGG	AAGGCCATGTATTCCGATACCGG	TTCGTGACATCAGATACGAGAGG	AGTGCTCGATAGACCAAGCAGGG
TCCCGATAAACACCTTCCTCCGG	TTGCAGCAGCTGGAGTAAGCGGG	CATCAAGGTTGACAAGGGTACGG	CGCTCCATCCTGTTGTCAAGAGG
GTGTTCTCGTTCATACATTCGGG	TCACAGTGGCCGAAAGCTTCTGG	CCTGGGTGAAAGTTTCGCCATGG	TCACCTTTGCAATTGGAGCAGGG
AACCCGATGAAACGGCGACAAGG	ATTGAGGATTGCGTAAGAGCTGG	GCGTCACTACTTTGGGCAGATGG	TTCCAAGGCAGGTGCCAATATGG
GTCGCTGGTGTGTAAATCGTGGG	GGCCTGCTGGGACTACAAATAGG	CGGATGTAGTATATGATGCCTGG	AGAGATTTGCACCCGCAATGAGG
TCACCCATTGTCCACACAAAAGG	TCTCCAATTGGCATCGCTTCCGG	TTACCATCTTGAGGTTGCTCTGG	TTAGTATTCAATCTTCCCCGAGG
CGTATAGCGTATGCATATTCTGG	GAGTCCTACAATATAGTACCGGG	GAGATTCAACAACACCAGGCAGG	CGGCTCCCTCTACACATCAAGGG
ATACGGTTTGTGGGATGCCAAGG	TTCAGCGTATCCATTTCCTTCGG	TTGGGGTTCCAGTTCCCGAAGGG	CCTCACCGCATCCAACTCTAGGG
ATCTGCTGATGAAGAAGAGATGG	CCAATCTGATTTCGCTGCTATGG	CGACAAGGGTCGGTTGTCTAAGG	GAGATGGAAAAGGCACTTACAGG
GCTGCGGAGCTTGAACCATAAGG	CAATCTGATTTCGCTGCTATGGG	CGTTCTTAATCGTCGCTCCGAGG	GGAAAAGGCACTTACAGGCTTGG
AGGGTCCTTTAGCCCATCAGAGG	AAGGTGGATACGGAAACGGTGGG	GTCTTCCAGTGACAAGTTCCCGG	CACCATCAGAGGATAACATGCGG
TTAGCCCATCAGAGGTTGCAGGG	GGGTATGGTGGAGGTGGATACGG	CGATTTGACGGTGCATATCATGG	TCTTTCCTCGGAACGGGAGATGG
ACTTGGACGAGTCAACTCGTCGG	CTTTGTCTTCGCCATACTTCCGG	TCCAGATAGCCACAGTGAGGGGG	ATGGCTCGAGTTTGTCTCTGAGG
TGGACACGGTAGAAGACAAGGGG	AGCATGACCTGTTTCTTGCACGG	CACTCTTCGATGGTGCCGATGGG	CATGTGGGATGGACTCCACAAGG
ATGCATAATGGGGCGCCACAGGG	CTGAAGATTGCAACCCAGACGGG	TCCAGGGAGCAAAAAGATTGTGG	ATGGATGGTTGCCAGTAGCACGG
TGCATGTTGCAGCGACTGCTTGG	GCATTCCGATTTCCATCGCTTGG	CGCCAATGGCTCGAATCACAGGG	AACTCGACGCTGCATAAGCTGGG
TGAAGAGCAAGTAGCCATAGTGG	GTCCCAAAGTCCAGGGTTCAGGG	CTTCCTCATCCAAAGTACCAGGG	GACACTACACGATCAGTACCCGG
TTGTTGACGAACGCAAGCCTTGG	GAAACTATTGTCGAAGCAACTGG	CCCTGTCATTTAAAGGCTTGAGG	ATACCGATCCGTACTGAATTTGG
CTGTAAAGAACAAAAGGCCACGG	AGTTTCTATCGTCAGTTCCCTGG	CAACTTGGCATTATCAGACGTGG	CTCCATATCTCCGGTTGTCTCGG
ATTTGCAGTGTCGTCTTTGGAGG	CCCAAGAGATCCCATCACTAAGG	ATCATAGACGTACTCGGCGGAGG	ACACCATTTGCCATTCACAATGG
TAAAGCTATCTCTCACACCCGGG	TGTCAAGCGTGTGTCACCTTTGG	AGCTGAGAAATTAGAGCCGCTGG	TCACCATTGTGAATGGCAAATGG

GCCACTCTGGATGAAAGCTGTGG	ATTTCTGTCCGAGTATCGTAGGG	TGAGAAATTAGAGCCGCTGGTGG	ATCGGTTGTTGACAAGTTTCAGG
GTCTGAAGAGAAAGAGGTAGAGG	CATATGTGAGTGAACCACCTGGG	TTCGATGTCGAAGTCCTTGTAGG	TCTCTGCATTCGCTACTCCTGGG
CACCCCAATCTTGGAGTTGCTGG	CAGAAAGACGAAATCAACGGAGG	ATGATTGGGCCACAAGGGGTGGG	GAGACTACCACACTCGTCTCAGG
CTTGGAGTTGCTGGATTCTAGGG	CCAGTGTGTATTGAGTATTGCGG	GCTAGTCCCTGGAGATTCTGCGG	AATCTACGGTAGAGGCTGCTAGG
ATGCCTGTCACTGCTGAAGTTGG	CCTCCTGCAATTCACTTTGATGG	TGTGGCAATGAATGGGGCTTTGG	GACAACGTAGTAGAACAGCGAGG
CTCCCTGTGGATAAAGACGGGGG	TTAACATGGAGGCAAATCCTTGG	GTAACCCTAGAGCTCTTAGAAGG	ACAGAGGAATTGTTATCAGCTGG
GAAGGTATTGAATTCCGGGGTGG	TAACATGGAGGCAAATCCTTGGG	TCAACCTTCTAAGAGCTCTAGGG	GGTTGTGGGATTAGCAGATGTGG
CATTAACACTGCCGTACCCGCGG	AATCTGATTGACCCGTCAGGAGG	GTTCATTTCCTCATTGAGGTTGG	CTTAGGAAATCCGCTTCGTTCGG
TCCAAGGCCTTAAGCAATTCTGG	TTGCAACCCAACCTCCTGACGGG	AATGCGCTCCAACCTCAATGAGG	AGGAAACAATCCTACTCGTTCGG
AGGATCCTGGTAAGCCTAGCCGG	TTCAGGCAAGCGTTAACAGAAGG	CGAGCTCGATTTCGGTAGGATGG	CCTCTAGCTGCAATACTGGCCGG
TGTCGATGGTGCTTCTTCCCTGG	GGCGATCCCGGAGAAATTTATGG	AACACGAAACCGTGAACGAAAGG	ACCGGAAATATCATCACCATCGG
GCTTCTTCCCTGGTTCTCACTGG	ATTTGGTGGTACCCAAAAACGGG	AACACTCCAATGTTGCCATCAGG	TCCGATGGTGATGATATTTCCGG
TTAAAACACCACGGTGCGTAGGG	GGCGGTCAAGTCATGTGAAGTGG	CCAAACCATCGGTCAATCCAAGG	AAGAAAAGGGCTTGGTGTAGCGG
GGACGGATGGGAAAGTGATGCGG	CGTTTGCTCATCCATCAACAAGG	GGTTGGGTGATCTTGCTCAGAGG	GTACAGTTACTCTGTTATGGAGG
GGCTACTTATGTCGTGCTCCCGG	ATCTTCTCAGCCACTTTCTAAGG	AGGAGACTGAGATGGTGCAATGG	AAATTGCGAAGTTCAACCGTCGG
CCCAAACTTGCTCGAACGTTCGG	TCAATGGGGGGTTAATGCACAGG	GGTGGCTGTGAAGATAATGAAGG	CGTAAACCGAAGCAACTCTTGGG
ACAAGCTGGATAAACGGACATGG	CAATGGGGGGTTAATGCACAGGG	TTTCTGCAACTGTTCCAGCTCGG	CGAATTGCGCCATCTTGTTGGGG
CGTCTTATTAACTATGCCGAAGG			

Table S4. Sequences of barcoded primers.

	Forward primers		Reverse primers
GACgcctagTATAAGCGAAAGAAGCATCAGA	GACgacgtaTATAAGCGAAAGAAGCATCAGA	CGAgtcacgTATAAGCGAAAGAAGCATCAGA	GACacgtcaGACCCGAATTTGTGGACC
GACgcgctaTATAAGCGAAAGAAGCATCAGA	GACgactgtTATAAGCGAAAGAAGCATCAGA	CGAgtcgctTATAAGCGAAAGAAGCATCAGA	GACaccatgGACCCGAATTTGTGGACC
GACgcggatTATAAGCGAAAGAAGCATCAGA	GACgagtcaTATAAGCGAAAGAAGCATCAGA	CGAgtcgtaTATAAGCGAAAGAAGCATCAGA	GACacgagcGACCCGAATTTGTGGACC
GACgcgtcaTATAAGCGAAAGAAGCATCAGA	GACgatcgaTATAAGCGAAAGAAGCATCAGA	CGAgtggacTATAAGCGAAAGAAGCATCAGA	GACacagcgGACCCGAATTTGTGGACC
GACgctacgTATAAGCGAAAGAAGCATCAGA	GACgcaccgTATAAGCGAAAGAAGCATCAGA	CGAgacatgTATAAGCGAAAGAAGCATCAGA	GACatgatgGACCCGAATTTGTGGACC
GACgtacgaTATAAGCGAAAGAAGCATCAGA	CGAgcctagTATAAGCGAAAGAAGCATCAGA	CGAgacgtaTATAAGCGAAAGAAGCATCAGA	GACatcatcGACCCGAATTTGTGGACC
GACgtcacgTATAAGCGAAAGAAGCATCAGA	CGAgcgctaTATAAGCGAAAGAAGCATCAGA	CGAgactgtTATAAGCGAAAGAAGCATCAGA	GACatggtcGACCCGAATTTGTGGACC
GACgtcgctTATAAGCGAAAGAAGCATCAGA	CGAgcggatTATAAGCGAAAGAAGCATCAGA	CGAgagtcaTATAAGCGAAAGAAGCATCAGA	GACatgctgGACCCGAATTTGTGGACC

GACgtcgtaTATAAGCGAAAGAAGCATCAGA	CGAgcgtcaTATAAGCGAAAGAAGCATCAGA	CGAgatcgaTATAAGCGAAAGAAGCATCAGA	GACagcacgGACCCGAATTTGTGGACC
GACgtggacTATAAGCGAAAGAAGCATCAGA	CGAgctacgTATAAGCGAAAGAAGCATCAGA	CGAgcaccgTATAAGCGAAAGAAGCATCAGA	GACagctcaGACCCGAATTTGTGGACC
GACgacatgTATAAGCGAAAGAAGCATCAGA	CGAgtacgaTATAAGCGAAAGAAGCATCAGA		GACagatgtGACCCGAATTTGTGGACC
			GACagacgaGACCCGAATTTGTGGACC

Table S5. List of primers used in this study.

**Purpose**	**Names**	**Primer sequence (5’-3’)**
**RT-PCR**	GhMLP423-F	5’ GATTTCGAGCTCAAGTCTCCTGC 3’
	GhMLP423-R	5’ TCATCCACTGATTCGATTCTCTC 3’
	GhEps15-F	5’ TGGAACAGGGGGGCAGTG 3’
	GhEps15-R	5’ ACCCGTGGGAAGCTCAAT 3’
	GhPR1-F	5' CAATGGACCTTACGGGGAGAAT 3’
	GhPR1-R	5' GCACCCGACTCTCTTGGTTCTG 3’
	GhPR2-F	5' CCACCAGCAGCAGAAGTTATCG 3’
	GhPR2-R	5' TTCAAGGTTTGCACTCGGAAGA 3’
	GhPR5-F	5’ GGACCATCGATGTGCCTGC 3’
	GhPR5-R	5’ TCAATGCAAAGGTTGGGGTG 3’
	AtPR1-F	5’ CAGTGAGACTCGGATGTGC 3’
	AtPR1-R	5’ TATGATGCTCCTTATTGAAATAC 3’
	AtPR2-F	5’GCAGCATTGGAGAAATCAG 3’
	AtPR2-R	5’ TCTCATCGAACATAGCGAAT 3’
	AtPR5-F	5’ GCTACGCTTATGACGACGAAAC 3’
	AtPR5-R	5’ CATAGTCATCAGCTTTCCTTAT 3’
**VIGS**	Eps-TRV-F	5' ATGGCGGGGCAAACTCA 3'
	Eps-TRV-R	5' TCATACCAGCATTTGGAACTCC 3'

	MLP423-TRV-F	5’ CCATGAATCAAACAAAAATGC 3’
	MLP423-TRV-R	5' GGAGATCCCCATCAATG 3'
